# MiRInter-Trans: a transformer-based framework for microRNA interaction prediction

**DOI:** 10.1093/bioadv/vbag073

**Published:** 2026-03-09

**Authors:** Marco Nicolini, Federico Stacchietti, Francisco Javier Molina, Carlos Cano, Jesus Alcala-Fdez, Alberto Paccanaro, Elena Casiraghi, Giorgio Valentini

**Affiliations:** AnacletoLab—Dipartimento Informatica, Università degli Studi di Milano, Via Celoria 18, Milano (MI) 20133, Italy; AnacletoLab—Dipartimento Informatica, Università degli Studi di Milano, Via Celoria 18, Milano (MI) 20133, Italy; Department of Computer Science and Artificial Intelligence, University of Granada, Periodista Daniel Saucedo Aranda, Granada 18071, Spain; Department of Computer Science and Artificial Intelligence, University of Granada, Periodista Daniel Saucedo Aranda, Granada 18071, Spain; Department of Computer Science and Artificial Intelligence, University of Granada, Periodista Daniel Saucedo Aranda, Granada 18071, Spain; Andalusian Research Institute in Data Science and Computational Intelligence (DaSCI), University of Granada, Avenida del Conocimiento 41, Granada, 18016, Spain; Instituto de investigación Biosanitaria ibs. GRANADA, Av. de Madrid, 15, Granada, 18012, Spain; Escola de Matemática Aplicada, Fundacao Getulio Vargas, Rio De Janeiro, 22250-900, Brazil; Department of Computer Science, Bioinformatics Centre for Systems and Synthetic Biology, Royal Holloway, University of London, Egham TW20 0EX, London, UK; AnacletoLab—Dipartimento Informatica, Università degli Studi di Milano, Via Celoria 18, Milano (MI) 20133, Italy; ELLIS—European Lab for Learning and Intelligent Systems, Milan Unit, Milano (MI), 20133, Italy; Department of Computer Science, Aalto University, Tietotekniikantalo, Konemiehentie 2, Espoo, 02150, Finland; Environmental Genomics and Systems Biology Division, Lawrence Berkeley National Laboratory, 1 Cyclotron Road, Berkeley (CA) 94720, USA; AnacletoLab—Dipartimento Informatica, Università degli Studi di Milano, Via Celoria 18, Milano (MI) 20133, Italy; ELLIS—European Lab for Learning and Intelligent Systems, Milan Unit, Milano (MI), 20133, Italy

## Abstract

**Motivation:**

The accurate prediction of microRNA (miRNA) interactions represents a significant challenge in computational biology, with far-reaching implications for understanding gene regulatory networks and developing RNA-based therapeutics. We present *miRInter-Trans*, an innovative computational framework that synergizes a pre-trained RNA foundation model (RNA-FM) with a feed-forward neural network to predict miRNA interactions using solely sequence information. Our approach harnesses the power of transformer-based embeddings to capture intricate sequence patterns and potential structural motifs without relying on handcrafted features or thermodynamic parameters.

**Results:**

Through comprehensive evaluation across three distinct interaction types (miRNA-lncRNA, miRNA-miRNA, and miRNA-snoRNA), we demonstrate that *miRInter-Trans* outperforms traditional Minimum Free Energy methods and other recently proposed computational approaches, achieving AUROC above 0.9 in both the miRNA interactions datasets used in our experiments. Notably, our models can accurately perform “de novo” miRNA interaction prediction, i.e. prediction of interactions for miRNA for which no or very reduced interaction data are available.

**Availability and implementation:**

The implementation and datasets used in this study are available at https://github.com/AnacletoLAB/miRInter-Trans.

## 1. Introduction

Deciphering the complex network of RNA–RNA interactions is fundamental for understanding the regulatory mechanisms that govern cellular processes (Hombach and Kretz 2016). Among non-coding RNAs (ncRNAs), microRNAs (miRNAs) play particularly crucial roles in post-transcriptional regulation through interactions with diverse RNA species. However, experimental characterization of these interactions remains challenging due to technical limitations, high costs, and the dynamic nature of RNA complexes (Winkle *et al*. 2021).

Early computational approaches relied primarily on thermo-dynamic modeling and accessibility calculations. Tools such as IntaRNA (Mann *et al*. 2017) employ detailed energy models that account for both duplex formation and the energetic cost of exposing binding regions. While highly effective in bacterial systems (Umu and Gardner 2017), these methods often struggle in eukaryotic contexts due to oversimplified energy assumptions, difficulties in parameterization, and the inability to capture non-canonical interactions.

Beyond physics-based models, network-based methods have recently been proposed (Torgano *et al*. 2025). For example, the SPMLMI framework (Xu *et al*. 2021) formulates miRNA–long non-coding RNA (lncRNA) interaction prediction as a link prediction task on a bilayer network that integrates miRNA–miRNA similarity, lncRNA–lncRNA similarity, and validated miRNA–lncRNA interactions. This approach leverages the principle of “structural consistency” (Lü *et al*. 2015), which assumes that biological networks contain hidden organizational patterns preserved under perturbation. Although effective when sufficient interaction data are available, network-based models remain constrained by their reliance on existing annotations and limited generalizability to unseen RNAs.

The advent of deep learning offers a new paradigm for RNA interaction prediction. In particular, transformer-based foundation models trained on large RNA sequence corpora have been shown to capture biologically meaningful representations directly from raw sequences (Chen *et al*. 2022, Sapoval *et al*. 2022, Valentini *et al*. 2023), identifying motifs and long-range dependencies without requiring handcrafted features or structural information. Recently, several deep-learning based methods have been proposed to predict miRNA-lncRNA interactions, including graph convolution network with conditional random field (Wang *et al*. 2022), feature embedding and deep feature mining methods (Yu *et al*. 2022), hybrid methods that combine bi-directional recurrent neural networks and random forests (Kang *et al*. 2020), heterogeneous graph neural network (Li *et al*. 2025) and the combination of a Transformer Encoder with convolutional neural networks (Yang *et al*. 2025). A recent survey on computational methods for miRNA-lncRNA interaction prediction classifies them into two main categories: network-based and sequence based (Sheng *et al*. 2023). Network-based methods firstly construct separate miRNA and lncRNA similarity networks using expression profiles or sequence information and then connect miRNA and lncRNA using interaction data from public databases. These methods then train machine learning tools on the overall integrated network to predict mirRNA-lncRNA interactions using graph based diffusion methods (Huang *et al*. 2018), embedding techniques based on first and second order random walk followed by boosting ensemble methods (Zhao *et al*. 2020), graph convolutional neural networks and conditional random fields (Wang *et al*. 2022), or graph attention networks and counterfactual links to construct embeddings used as input to a multi-layer perceptron for the final prediction (He *et al*. 2025). Other methods, like SPMLMI (Xu *et al*. 2021) and SPCMLMI (Wang *et al*. 2022) formulates miRNA–long non-coding RNA (lncRNA) interaction prediction as a link prediction task on a bilayer network that integrates miRNA–miRNA similarity, lncRNA–lncRNA similarity, and validated miRNA–lncRNA interactions. This approach leverages the principle of “structural consistency” (Lü *et al*. 2015), which assumes that biological networks contain hidden organizational patterns preserved under perturbation. A method called MPGK-LMI predicts miRNA-lncRNA interactions based on known miRNA-lncRNA interactions and Gaussian kernel similarity, using graph attention networks to aggregate the resulting similarity matrices and a classifier module to score the final predictions (Xie *et al*. 2024).

Sequence-based methods only use lncRNA and miRNA sequence information to train deep learning methods for predicting interactions (Sheng *et al*. 2023). In Zhang *et al*. (2020), an ensemble of deep learning models based on convolutional neural networks and recurrent neural networks was proposed to predict miRNA-lncRNA interactions in plants. Another approach integrates sequence-based and manually extracted features to train a hybrid deep learning system with convolutional neural networks, bidirectional gated recurrent units and random forests combined with fuzzy decision for the final prediction (Kang *et al*. 2020). Deep learning methods are also the core of preMLI, that applies RNA2vec embeddings (Yi *et al*. 2020) to train convolutional neural networks, bidirectional gated recurrent units and attention layers to uncover potential miRNA-lncRNA interactions (Yu *et al*. 2022)

Both network and sequence base methods present several limitations. Indeed, network-based methods share the problem of constructing real world heterogeneous graphs that are the learning objects of the machine learning methods used to make inferences on the interactions. Moreover, expression profiles and functional information used to construct miRNA and lncRNA similarity networks tend to have high noise, incompleteness and imprecision and models needs to deal with both the noise of the data and the noise of the manually constructed graphs (He *et al*. 2025). On the other hand sequence-based methods like convolutional neural networks often struggle in detecting long range interactions and recurrent neural networks, due to their sequential learning nature, tend to forget initial nucleotide information during the sequential processing of the RNA molecule (Pascanu *et al*. 2013).

Moreover, these methods are limited to the prediction of miRNA-lncRNA interactions and cannot be directly applied to other types of RNA molecules.

To overcome these limitations, we introduce *miRInter-Trans*, a sequence-based transformer-based model able to represent and predict novel interactions for different types of miRNA interactions not only limited to miRNA-lncRNA, but extended also to miRNA-miRNA and miRNA-snoRNA (small nucleolar RNA) in a unified framework. In our implementation we specifically fixed RNA-FM (Chen *et al*. 2022) to generate RNA sequence representations using a single embedding backbone based on a foundational model, and to improve performance we evaluated key downstream design choices, including different strategies to convert nucleotide embeddings into sequence-level representations and data augmentation strategies.

A preliminary version of *miRInter-Trans* has been presented at the the IWANN2025 conference (Nicolini *et al*. 2025).

Our contributions are three-fold:

- The first transformer-based approach for general miRNA–ncRNA interaction prediction that operates solely on sequence embeddings, removing the need for structural or manually engineered features.
- The first model capable of obtaining accurate results on “de novo” predictions of miRNA interactions, i.e. capable of predicting interactions for miRNAs for which very few or no interactions are available a priori.
- Experimental evaluation on two publicly available datasets, demonstrating that *miRInter-Trans* significantly outperforms traditional energy-based models and network-based state-of-the-art methods.

## 2. Methods

### 2.1. Datasets

Our study relies on two datasets of miRNA–ncRNA interactions. The first dataset is the *RNA-KG interaction dataset*, which includes three biologically relevant interaction types: miRNA–lncRNA, miRNA–miRNA, and miRNA–snoRNA. These interactions are derived from RNA-KG, a manually curated knowledge graph of RNA interactions (Cavalleri *et al*. 2024). We selected this dataset both for its reliability and for the diversity of interaction types it encompasses.

The second dataset consists exclusively of experimentally validated lncRNA-miRNA interactions and was obtained from the lncRNASNP database (Miao *et al*. 2018). This dataset not only provides a comprehensive set of validated lncRNA–miRNA interactions but also enables direct comparison with the SPMLMI method (Xu *et al*. 2021), which was benchmarked on the same data. For both datasets, interactions are physical. These include direct binding as well as regulatory effects that are mediated by RNA–RNA binding. In RNA-KG, such interactions may derive from either experimental evidence or curated computational predictions.

The two datasets differ markedly in both size and data type composition: lncRNASNP focuses on a single interaction type (miRNA–lncRNA) and provides a much larger number of validated positives (about 16 000), whereas RNA-KG includes three heterogeneous interaction types (miRNA–lncRNA, miRNA–miRNA, miRNA–snoRNA) and, for the miRNA–lncRNA subset specifically, contains only about 2000 positives.

Further details on the two datasets are provided below.

#### 2.1.1. RNA-KG interaction data

The RNA-KG dataset initially contains 81 891 miRNA–lncRNA pairs, 2071 miRNA–miRNA pairs, and 2039 miRNA–snoRNA pairs. To comply with the sequence length constraint of RNA-FM (maximum 1022 nucleotides), we applied a filtering procedure that retained only molecules within this limit. After filtering, the dataset comprises 13 104 unique interaction pairs: 9058 (11.1%) miRNA–lncRNA, 2071 (100%) miRNA–miRNA, and 1975 (96.8%) miRNA– snoRNA interactions. These involve 3033 unique RNA sequences: 688 lncRNAs, 1900 miRNAs, and 445 snoRNAs.

#### 2.1.2. LncRNASNP interaction data

The lncRNASNP database (Miao *et al*. 2018) provides a curated collection of experimentally validated lncRNA–miRNA interactions [NONCODE lncRNA IDs (Fang *et al*. 2018) versus miRNA IDs from miRBase (Kozomara *et al*. 2019)].^1^ Since raw sequences are not distributed directly, we retrieved human lncRNA sequences from GENCODE (Mudge *et al*. 2025) and miRNA sequences from miRBase.

The original dataset contains 39 366 lncRNA–miRNA pairs involving 3150 lncRNAs and 262 miRNAs. Filtering out lncRNAs longer than 1022 nucleotides removed 1483 sequences, yielding a final set of 16 018 validated interactions, covering 1667 lncRNAs and 262 miRNAs.

For benchmarking, we also downloaded the miRNA and lncRNA similarity networks used in SPMLMI (Xu *et al*. 2021) from the authors’ GitHub repository.^2^

### 2.2. Data augmentation

To improve model robustness and data efficiency in a setting where experimentally validated interactions are limited, we applied a data augmentation strategy that generates multiple, equivalent views of each interaction pair. Importantly, this strategy does not create synthetic interactions; it only produces different representations of the same labeled pair.

Let the set of length-filtered RNA molecules be $R=\{r_{i}\},i=1,\ldots ,|R|$, and let ϕ:R→T be a type mapping where T={miRNA,lncRNA,snoRNA}. Since the label is assigned to the interaction pair independent of the molecule order in the pair, we treat each interaction as unordered: $\left(r_{i},r_{j})\equiv (r_{j},r_{i}\right)$.

We also define a sequence-reversal operator F(·) such that, for an RNA sequence $r=(n_{1},\ldots ,n_{L})$, its reversed form is $r^{F}=F(r)=(n_{L},\ldots ,n_{1})$.

Augmentation strategy.

For each pair $\left(r_{i},r_{j}\right)$, we generate the following four views:

1. *Original pair:* $\left(r_{i},r_{j}\right)$.
2. *Order-reversed pair:* $\left(r_{j},r_{i}\right)$. This enforces that the predictor does not depend on an arbitrary ordering of the two molecules in the input.
3. *Sequence-reversed view:* $\left(r_{i}^{F},r_{j}^{F}\right)$, where each RNA is read in the opposite direction.
4. *Combined augmentation:* $\left(r_{j}^{F},r_{i}^{F}\right)$ that combines order reversed and sequence reversed pairs.

For compactness, we denote the augmented set associated with the positive pair $\left(r_{i},r_{j}\right)$ as


$$A(r_{i},r_{j})=\{(r_{i},r_{j}),(r_{j},r_{i}),(r_{i}^{F},r_{j}^{F}),(r_{j}^{F},r_{i}^{F})\}.$$

This procedure increases the number of available instances by a factor of four while preserving the original interaction set. Note that, at test time, augmented positive views are evaluated as distinct instances (see Section 2.5.1).

We emphasize that the sequence-reverse ordering operation is used *only* as a training and evaluation-time regularizer and does not imply that RNA function is orientation-invariant, nor does it aim to model folding or binding geometry. Rather, since RNA-FM produces position-dependent contextual embeddings, exposing the downstream predictor to both canonical and reversed-sequence views reduces spurious reliance on a single presentation convention allowing our models to learn from both sense and antisense sequences. In other words, from a biological standpoint, two interacting ncRNAs are antiparallel (5′–3′ vs 3′–5′), but from a ML standpoint we can use both the orientations to train the system, thus improving the cardinality of training data and the generalization performance of our model.

### 2.3. Negative sample generation

Besides existing (positive) interactions, supervised machine learning models need a set of negative (i.e. not-existing) interactions, i.e. pairs of molecules that do not interact. Since experimental resources typically provide validated positives but rarely provide confirmed non-interactions, we follow the *closed-world/weak-label* (Wang *et al*. 2017, Bahaj and Ghogho 2024) assumption commonly used in database-driven interaction prediction: observed interactions are treated as positives, while unreported pairs are treated as negatives for supervised learning. We note that “unreported” does not necessarily imply “non-interacting in vivo”; rather, it may reflect incomplete experimental coverage, thus potentially introducing false negatives. Nevertheless, this assumption enables a reproducible supervised setting consistent with prior work, and we mitigate leakage and sampling artifacts through strict split control and type-consistent negative sampling (described below).

Let  $P=\{(r_{i},r_{j})|\text{an}\;\text{interaction}\;\text{between}\;r_{i}\;\text{and}\;r_{j}\;\text{is}\;\text{observed}\}$ be the set of known positive interactions.

As defined in Section 2.2, each pair $\left(r_{i},r_{j}\right)$ is associated with an augmented set $A(r_{i},r_{j})$. To ensure that no augmented representation of a known positive can be sampled as a negative, we define the augmentation-aware positive closure $P_{\text{aug}}=\underset{(r_{i},r_{j})\in P}{\cup }A(r_{i},r_{j}),$ and we reject any candidate negative that matches an element of $P_{\text{aug}}$.

To avoid task-inconsistent negatives, we generate negatives by corrupting positive interaction pairs. Specifically, for each positive pair $(r_{i},r_{j})\in P$ (i.e. before augmentation), we randomly select one of the two molecules to replace and substitute it with a uniformly sampled RNA $r^{\prime }\in R$ of the same type. Without loss of generality, if $r_{j}$ is selected for replacement, we sample $r^{\prime }$ such that:

-
$r^{\prime }\neq r_{i}$ (no self-interactions);
-
$\phi (r^{\prime })=\phi (r_{j})$ (same molecular type as the replaced molecule);
-
$(r_{i},r^{\prime })\notin P_{\text{aug}}$ (not a known positive pair, including augmented representations).

From a statistical standpoint, negatives are generated by uniform sampling from the complement of the observed positives P (with the constraints described above), which corresponds to standard negative edge sampling in supervised link prediction. To reflect the natural imbalance between interacting and non-interacting pairs in biological systems, we generate a number of negative interactions that is $n_{\text{neg}}$ times larger than the number of positives examples |P|. In the RNA-KG experiments we set $n_{\text{neg}}=20$. For the LncRNASNP dataset we instead used all available negatives provided by the dataset, which results in $n_{\text{neg}}\approx 26$ and ensures a fair comparison with SPMLMI.

Finally, negative pairs are augmented to provide for each negative interaction also their order and sequence reversed views, using the same procedure employed to augment the positive interaction examples.

### 2.4. miRInter-Trans model architecture

**Figure 1:**
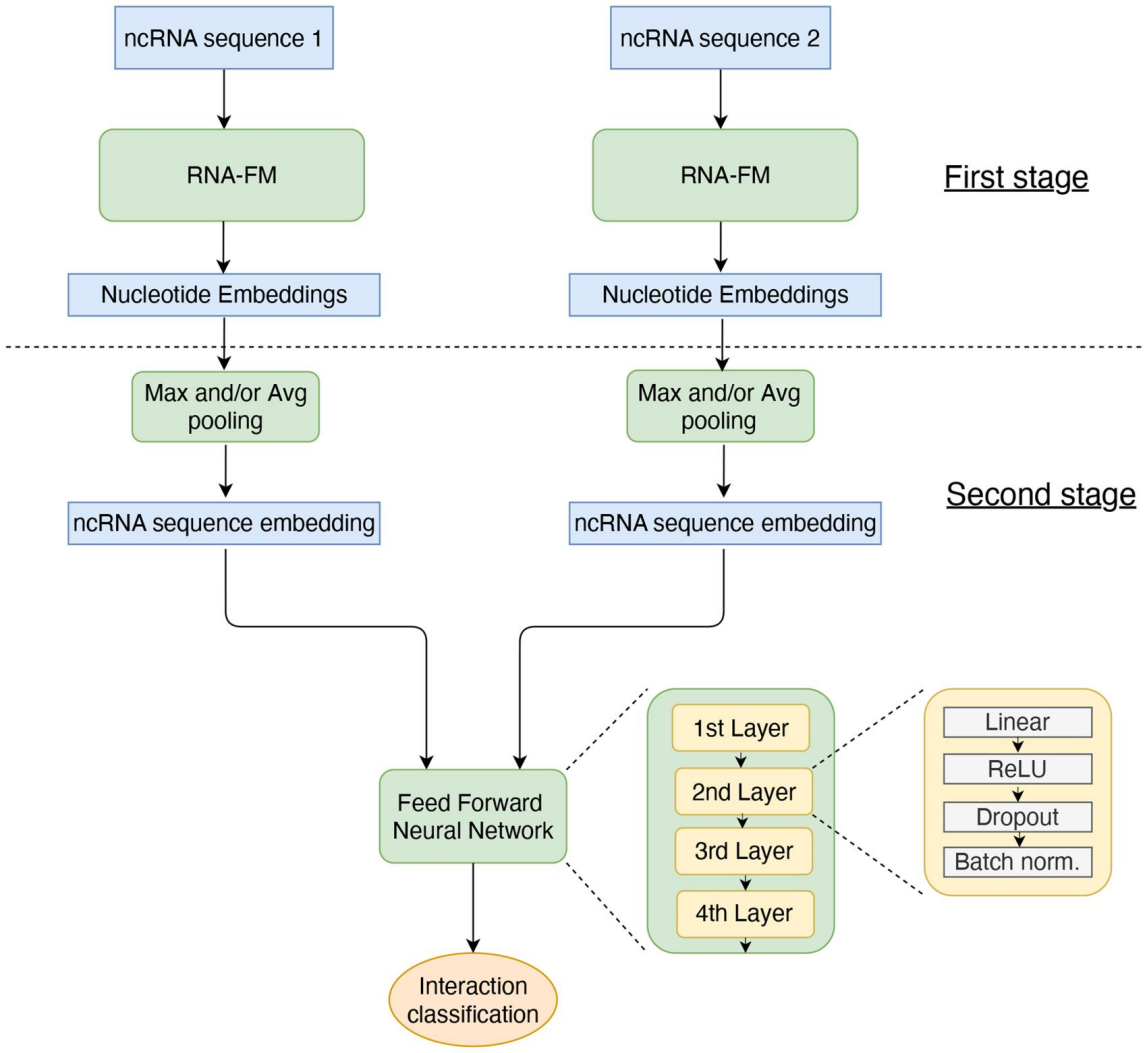
Architecture of *miRInter-Trans*. In the first stage RNA sequences are processed through the pre-trained RNA-FM model to generate contextual embeddings of the nucleotides. In the second stage, pooling operations are applied to create fixed-length embedded representations of the overall sequence of ncRNAs, which are finally processed by a feed-forward neural network for interaction prediction.

*miRInter-Trans* employs a two-stage architecture (illustrated in Fig. 1) that combines the representational power of RNA foundation models with a carefully designed prediction network.

#### 2.4.1. Embedding generation

**Figure 2:**
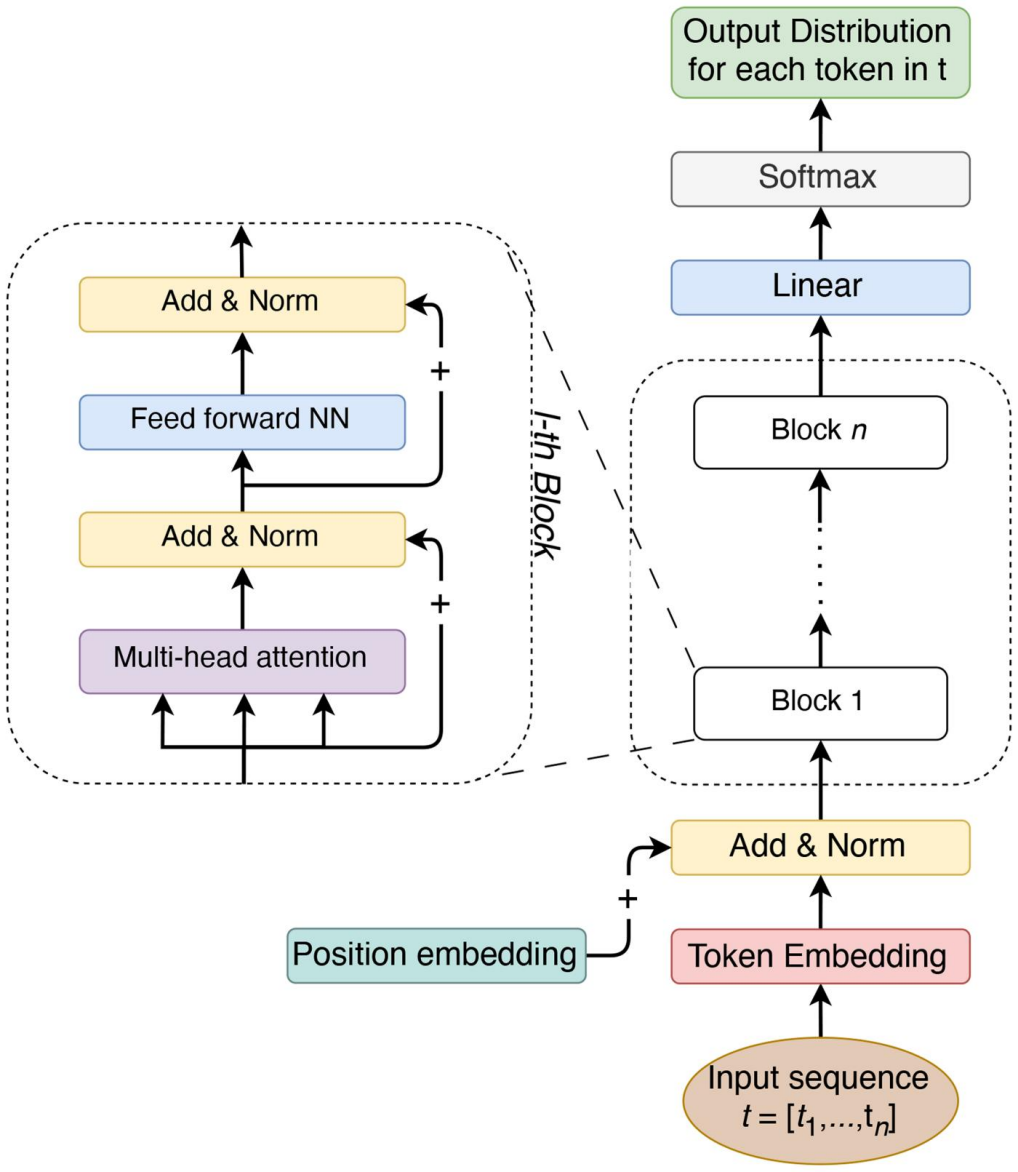
Architecture of RNA-FM, a BERT-like encoder transformer.

The first stage utilizes RNA-FM (Chen *et al*. 2022), a state-of-the-art BERT-like model (Devlin *et al*. 2019) pre-trained on over 23 million ncRNA sequences across 47 different databases, that is part of a larger model recently proposed for RNA 3D structure prediction (Shen *et al*. 2024). We selected RNA-FM because it provides RNA-specific representations while remaining computationally tractable for systematic evaluation of downstream design choices. For an input sequence of length *L* (with L∈[1,1022]), RNA-FM produces an L×d embedding matrix (d=640 is the embedding dimension), where each row corresponds to the contextual representation of a nucleotide in the sequence, since the tokenization process maps each nucleotide directly to a token. Figure 2 provides a schematic diagram of the RNA-FM BERT-based transformer, composed by 12 bidirectional encoder layers.

Having $h_{i}\in R^{d}$, that represents the $i^{th}$ row of the output embedding matrix $H\in R^{L\times d}$ of the RNA-FM model, we investigated three distinct strategies for converting these variable-length embeddings into fixed-size representations of the entire RNA sequences suitable for interaction prediction:

- **Average pooling**: Computes the mean across sequence positions: $e_{\text{avg}}=\frac{1}{L}\sum_{i=1}^{L}h_{i}$
- **Max pooling**: Captures the most salient features: $e_{\text{max}}=\max_{i=1}^{L}h_{i}$
- **Concatenated pooling**: Combines both average and max features: $[e_{\text{avg}};e_{\text{max}}]\in R^{1280}$.

For interaction pairs, we concatenate the pooled representations of both sequences, resulting in a 1280-dimensional vector for average/max pooling individually, or 2560-dimensional when using concatenated pooling. Supplementary Fig. S7 illustrates average and max pooling methods.

#### 2.4.2. Prediction network

The second stage consists of a fully connected feed-forward neural network comprising four layers with 1024 neurons each, ReLU activations, dropout with 0.2 rate, and batch normalization after every layer. A final linear unit with sigmoid activation produces the predicted interaction probability (Fig. 1).

#### 2.4.3. Mini-batch balancing

To mitigate class imbalance, we applied a mini-batch balanced learning strategy, by using a controlled ratio of positive and negative examples in each mini-batch. Let D be the complete dataset with *n* examples, where P and N denote the sets of positives and negatives, with $|P|=n_{p}$ and $|N|=n_{n}$, respectively. Each mini-batch *B* of size *m* is formed by randomly drawing $m_{p}$ positives (with replacement) and $m_{n}$ negatives (without replacement), such that $m_{p}+m_{n}=m$. The positive proportion is defined as


$$k=\frac{m_{p}}{m_{p}+m_{n}},$$

so that k=0.5 implies 50% of positives in the mini-batch. Positive samples are drawn with replacement due to their lower frequency, while negatives are sampled without replacement to ensure wider coverage of the available examples.

#### 2.4.4. Training protocol

For training *miRInter-Trans* we implemented a carefully optimized training procedure with the following parameters:

- Learning rate: $5\times 10^{-4}$ with cosine decay scheduling
- Batch size: 512 (with 30% positive and 70% negative examples randomly drawn in each mini-batch)
- Early stopping: 10 epochs patience
- Loss function: Binary cross-entropy
- Optimizer: Adam with weight decay

All experiments were conducted on a linux server equipped with 512 GB of RAM and an NVIDIA A100 GPU.The model was implemented in Python using PyTorch (v2.10). Training was performed under CUDA (v12.9). All experiments were executed with fixed random seeds.

### 2.5. Experimental settings

We considered two different experimental settings: five-fold cross-validation and “de novo” miRNA interaction prediction.

#### 2.5.1. Five-fold cross-validation for unbiased evaluation

Model generalization was assessed using five-fold cross-validation. Under this setting, interaction pairs in the test set were strictly disjoint from those in the training set, while allowing the same miRNA to appear in different interaction pairs across training and test folds.

To prevent augmentation-induced information leakage, we assigned folds at the level of the original interaction pair. Concretely, for each pair $\left(r_{i},r_{j}\right)$ we enforced that all elements of its augmented set $A(r_{i},r_{j})$ were assigned to the same fold. This guarantees that no augmented variant of a test interaction can appear in the training data.

For model selection, the training portion of each fold was further split into training (80%) and validation (20%) using the same unbiased splitting procedure (i.e. at the level of the original interaction pair).

#### 2.5.2. “De novo” miRNA interaction setting

To assess model generalization to previously unseen molecules, we adopted a scenario in which certain miRNAs were present exclusively in either the training or test set. This setting mimics a real-world scenario: we would like to predict interactions for a miRNA for which no interactions are known (“de novo” interaction prediction).

Given an interaction dataset, we first gathered all miRNAs appearing in any interaction pair and drew a random subset of these molecules. Each time a miRNA was selected, all interactions involving that miRNA, together with their augmented set to avoid leakage, were assigned to the test set. All remaining interactions formed the training set. This approach enforces that entire miRNA sequences are held out, ensuring that the model is tested only on molecules absent from training.

For both RNA-KG and lncRNASNP datasets, a unique “de novo” split was generated by randomly sampling 20% of the unique miRNA molecules and allocating all of their interactions to the test set, yielding an approximate 4:1 ratio between training and test interactions.

In the RNA-KG dataset, this procedure led to the following distributions of miRNA interactions in the the training and test sets:

- **miRNA–miRNA:** 206 miRNAs in the test set, 539 miRNAs in the training set, with 405 test interactions and 1666 training interactions.
- **miRNA–snoRNA:** 218 miRNAs in the test set, 431 miRNAs in the training set, with 401 test interactions and 1574 training interactions.
- **miRNA–lncRNA:** 454 miRNAs in the test set, 897 miRNAs in the training set, with 1833 test interactions and 7225 training interactions.

Supplementary Figure S6 shows the normalized histogram of the frequency of miRNA interactions in the “de novo” experimental setting for the training and test sets.

In the lncRNASNP dataset, the “de novo” split resulted in a test set comprising all interactions from 48 miRNAs, leaving 214 miRNAs in the training set, for a total of 3256 test interactions and 12 762 training interactions. Supplementary Figure S5 shows the corresponding normalized histogram of the frequency of miRNA-lncRNA interactions.

#### 2.5.3. Evaluation metrics

Model performance in both experimental settings was quantified using the Area Under the Receiver Operating Characteristic curve (AUROC) and the Area Under the Precision–Recall Curve (AUPRC). AUROC provides a global measure of discriminative power, while AUPRC is particularly informative for imbalanced tasks such as interaction prediction.

To provide a robust evaluation with augmented views, we treated each element of $A(r_{i},r_{j})$ as a distinct test instance and computed performance metrics over all these views, thereby explicitly assessing robustness to pair ordering and sequence-orientation conventions.

## 3. Results

**Figure 3:**
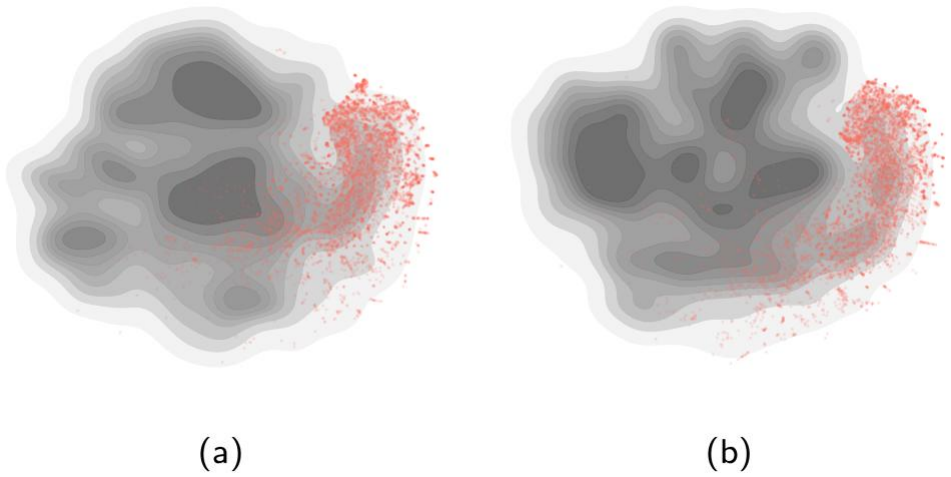
*miRInter-Trans* embeddings visualization. Two-dimensional t-SNE projections of the computed miRNA-lncRNA interaction embeddings obtained through (a) concat pooling, (b) MAX pooling on the RNA-KG test data. Negatives are represented using kernel density estimation lines in grey, while positives are plotted as red points.

Figure 3 reports two-dimensional t-SNE projections of the miRNA interaction embeddings extracted from the last hidden layer of the feed-forward prediction network of *miRInter-Trans* (Section 2.4.2 and Fig. 1). The 2D plot evidences that actual “positive” interactions are relatively well-separated from “negative” not-interacting pairs, supporting the use of RNA-FM pooled embeddings as input to the downstream feed forward network for interaction prediction.

The following sections report detailed results for both datasets and experimental settings, including comparisons with traditional thermodynamic approaches and state-of-the-art network-based methods.

### 3.1. miRNA interactions predictions using RNA-KG data

#### 3.1.1. Results on the five-fold cross-validation prediction scenario

**Figure 4:**
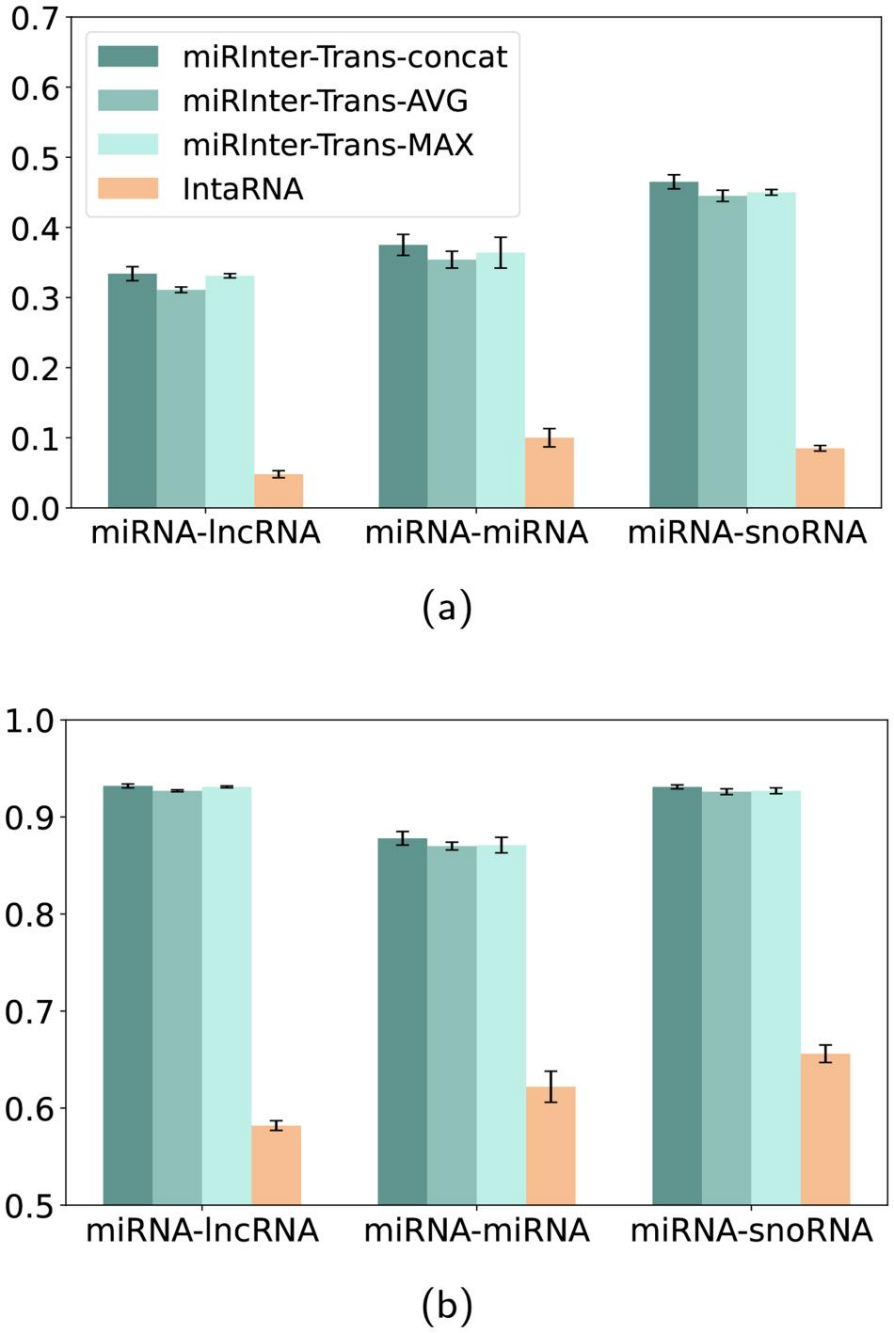
*miRInter-Trans* performance on augmented RNA-KG data across interaction types. (a) AUPRC, (b) AUROC. Error bars denote standard deviation across five folds.

In this experimental setting *miRInter-Trans* was evaluated on three distinct miRNA interaction prediction tasks by using both AUROC and AUPRC metrics. Figure 4 presents the results of *miRInter-Trans* on the three RNA interaction datasets (miRNA–lncRNA, miRNA–miRNA, and miRNA–snoRNA), using a five-fold cross-validation setting and data augmentation.

One-sided paired t-tests confirmed that *miRInter-Trans* was significantly better than IntaRNA, a thermodynamic-based model for RNA interaction prediction (Mann *et al*. 2017), in all datasets for both AUROC and AUPRC (*P* $<5\times 10^{-7}$ according to paired one-sided *t*-test, Fig. 4). Supplementary Tables S1 and S2 report detailed results obtained by *miRInter-Trans*, a random baseline and IntaRNA, as well results about their statistical comparison including also different *miRInter-Trans* pooling techniques.

**Figure 5:**
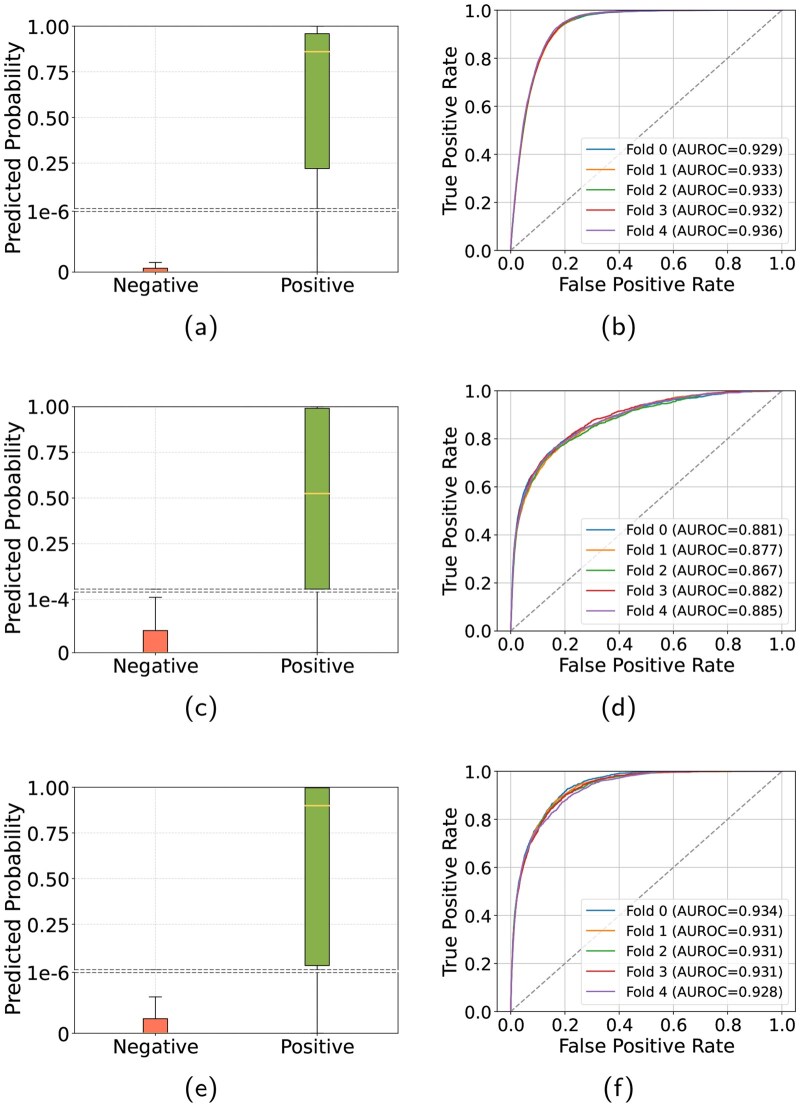
*miRInter-Trans* results on RNA-KG data with concat pooling: interaction prediction for miRNA-lncRNA pairs (top row), miRNA-miRNA pairs (central row), and miRNA-snoRNA interaction pairs (bottom row). (a, c, e): predicted probabilities for positive and negative examples across test folds. Log-scale is used in the continuous range $\left[0-1e^{-6}\right]$ to visualize low probabilities for negative interactions. (b, d, f) ROC curves on test folds.

The clear separation between positive and negative predictions (Fig. 5a, c, e) indicates that *miRInter-Trans* learns meaningful discriminative features for each miRNA interaction prediction task. The ROC curves (Fig. 5b, d, f) show that the proposed model can obtain accurate results on each fold. Supplementary Figure S2 reports plots of the precision recall curves and the confusion matrix for each interaction prediction task, confirming the prediction capabilities of the proposed model.

**Figure 6:**
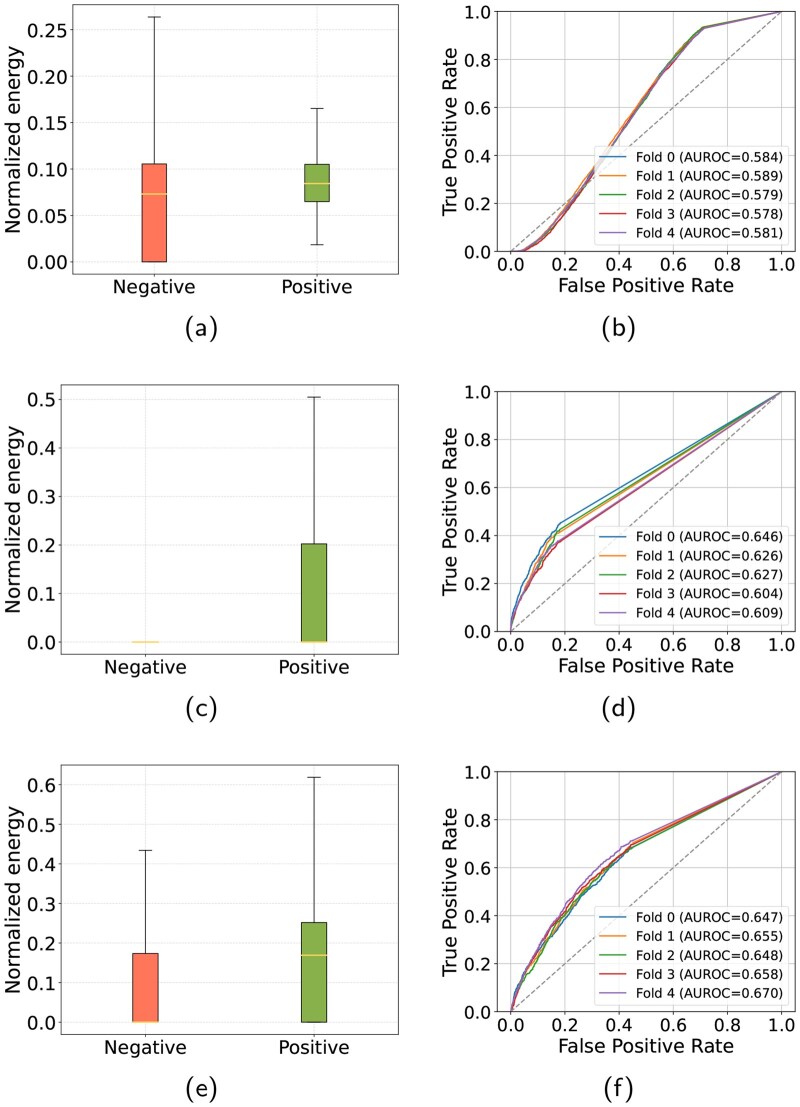
IntaRNA results on RNA-KG data: first row miRNA-lncRNA, second miRNA-miRNA and third miRNA-snoRNA interaction prediction. (a, c, e) Distribution of predicted probabilities for positive and negative examples across test folds (b, d, f) ROC curves on test folds.

IntaRNA achieved significantly worse results with respect to *miRInter-Trans* in terms of PRC and ROC curves and distribution of probabilities in positive and negative examples (Fig. 6 and Supplementary Fig. S1).

Finally, we note that data augmentation significantly improves *miRInter-Trans* results according to one-sided paired t-test (p<0.05, Supplementary Table S1). Furthermore, among the three pooling strategies (see Section 2.4.1), the concatenated pooling improves *miRInter-Trans* results according to one-sided paired t-test (p<0.05, Supplementary Table S1).

#### 3.1.2. Results on the “de novo” miRNA prediction scenario

**Figure 7:**
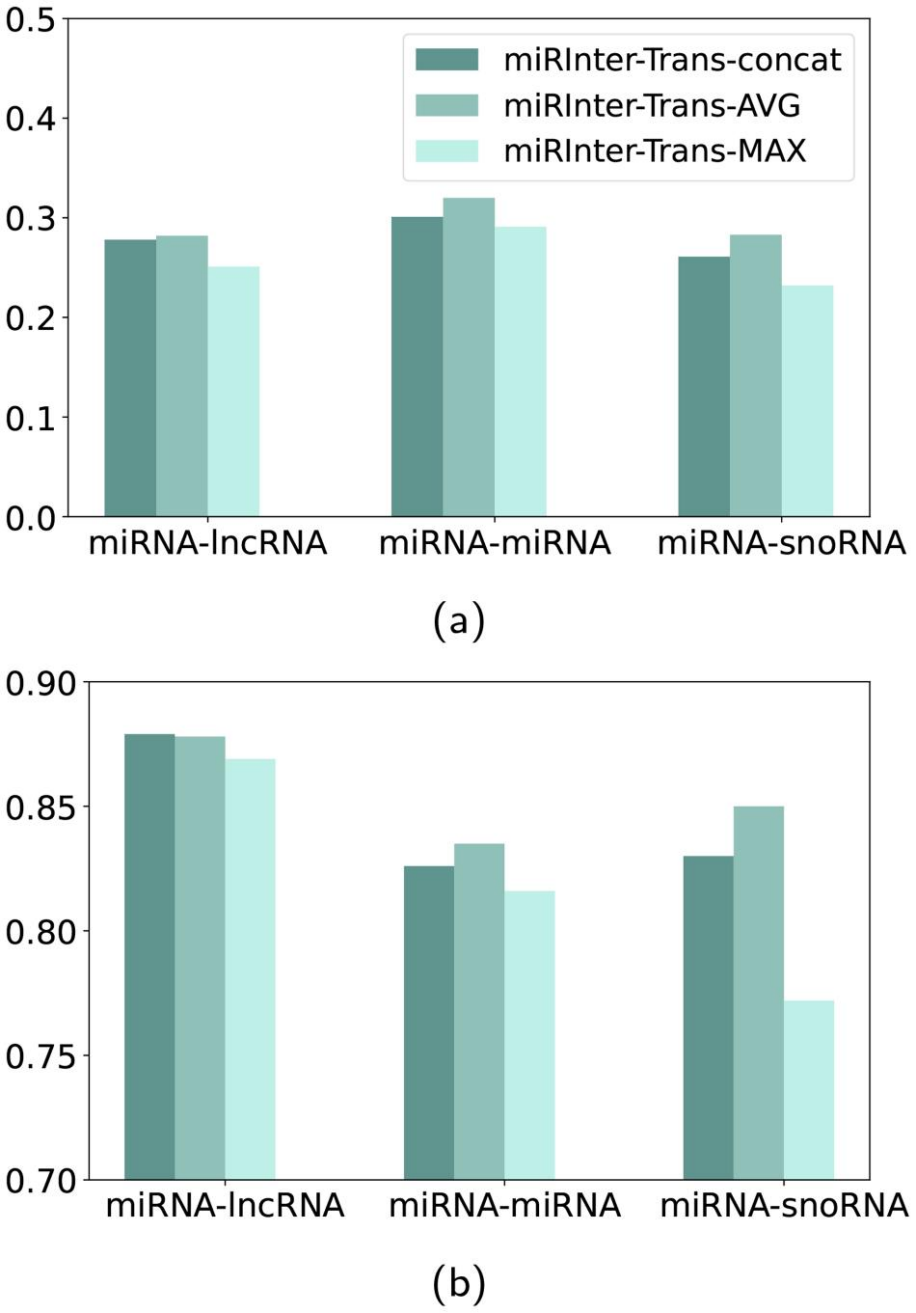
*miRInter-Trans* “de novo” *miRInter-Trans* miRNA interaction classification on the RNA-KG dataset, using different pooling techniques. (a) AUPRC, (b) AUROC.

Figure 7 shows that *miRInter-Trans* is able to obtain reasonable results also in this challenging setting, even if, as expected, with a certain decay in performance with respect to the five-fold cross-validation setting (Supplementary Table S3). Supplementary Figs. S3 and S4 show the detailed results in terms of ROC and precision-recall curves, confusion matrices and distribution of predicted probabilities in interacting and non interacting ncRNA pairs.

### 3.2. miRNA-lncRNA interaction prediction using lncRNASNP data

We tested *miRInter-Trans* on the lncRNASNP dataset (see Section 2.1), using the same experimental setting adopted for RNA-KG (five-fold cross-validation and “de novo”). We compared *miRInter-Trans* with SPMLMI, because SPMLMI is one of the state-of-the-art methods for the prediction of ncRNA interactions and was originally evaluated on the lncRNASNP dataset (Xu *et al*. 2021).

#### 3.2.1. Results on the five-fold cross-validation prediction scenario

By following the experimental settings of SPMLMI, the five cross-validation folds were generated by partitioning the known interactions of each miRNA into five subsets, ensuring that the relative proportion of miRNA interactions is preserved in each fold.^3^ We applied the same folds to test both SPMLMI and *miRInter-Trans*.

Supplementary Figure S8 shows the promising mean AUPRC (0.5667±0.0152) and AUROC (0.9458±0.0038) results achieved by SPMLMI across the five folds. The good performance obtained in the link prediction are supported by a relatively high structural consistency score $\sigma_{c}=0.566\pm 0.010$ (mean ± standard deviation) (Lü *et al*. 2015).

SPMLMI predicts links by perturbing the training network multiple times and reconstructing a perturbed adjacency matrix in the eigenbasis of the training network, as eigenvectors and eigenvalues capture the main structural patterns of the network. At each iteration, the algorithm removes a fraction of observed training links, projects the full training adjacency matrix onto the eigenvectors of the perturbed network, adjusts eigenvalues while keeping eigenvectors fixed, and reconstructs the perturbed training adjacency matrix. Averaging the resulting reconstructions emphasizes edges that consistently reappear due to structural regularities, which are therefore likely to correspond to true but hidden interactions.

It should be noted that, in this framework, all negative edges (non-interactions) remain fully observed during training, while only a subset of positive edges is hidden in the probe set. As a consequence, evaluation metrics such as AUROC and AUPRC may appear optimistic. This characteristic is not specific to SPMLMI but is a common feature of structural link prediction methods, which inherently exploit both the presence and absence of edges in the adjacency matrix to recover missing links.

**Figure 8:**
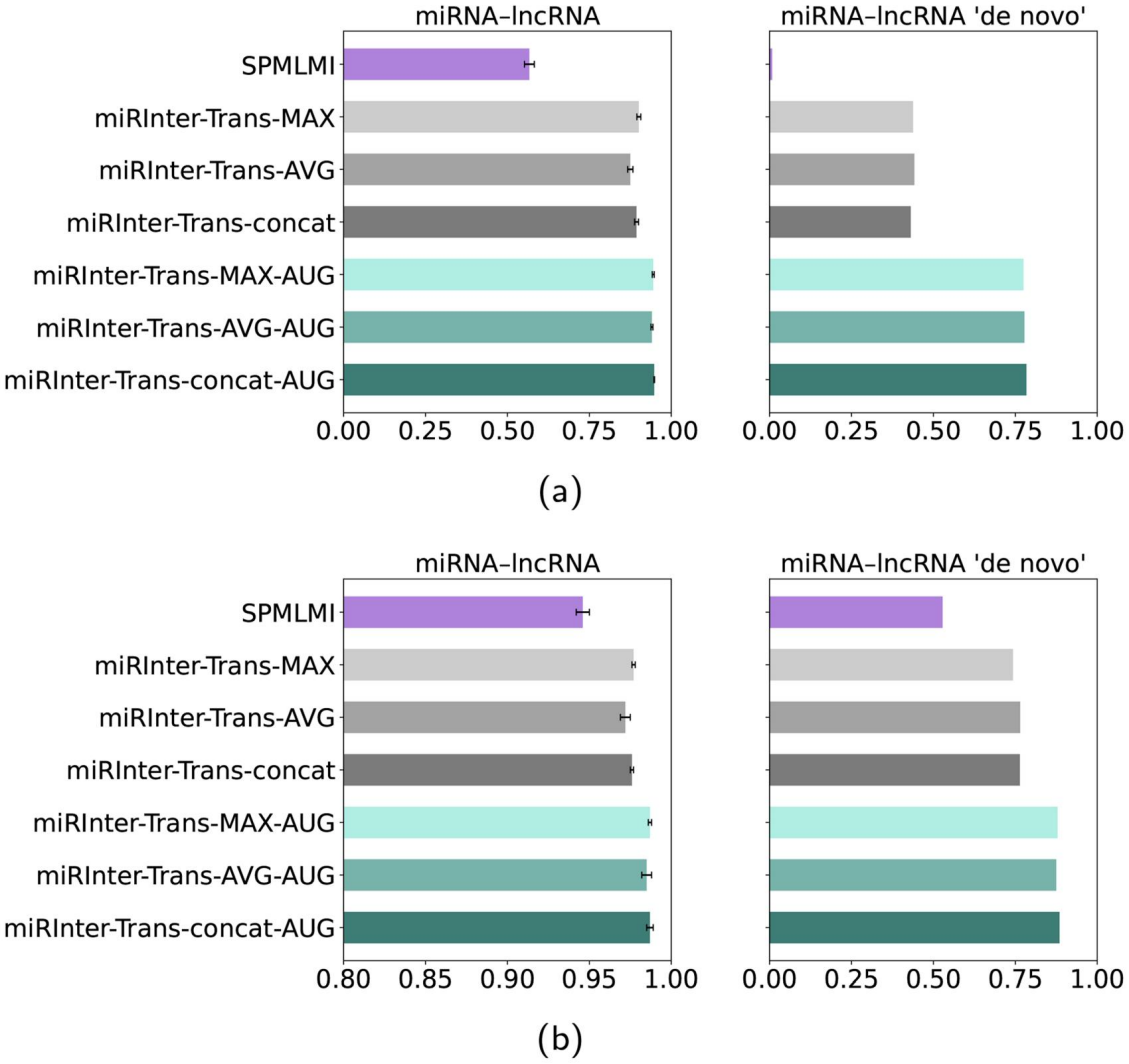
Comparison of *miRInter-Trans* and SPMLMI results on the lncRNASNP dataset, using the five-fold cross validation and “de novo” experimental setting. Bars on top represent the standard deviation. (a) AUPRC, (b) AUROC.

Figure 8 shows that *miRInter-Trans* consistently outperforms SPMLMI, with higher AUPRC values in the cross-validation setting (Supplementary Tables S5 and S6). Data augmentation significantly improves *miRInter-Trans* results (Table S4): according to the one-sided paired t-test, results are consistently and significantly higher when training with augmented data (p<0.05).

This is not surprising since the enrichment of new positive examples can improve the learning processes of neural networks, especially when the number of available examples is relatively small with respect to the cardinality of the learning parameters.

Finally, among the three pooling strategies (see Section 2.4.1), the concatenated pooling improves *miRInter-Trans* results (one-sided paired t-test, p<0.05, Supplementary Table S5).

#### 3.2.2. Results on the “de novo” miRNA prediction scenario

In this setting, the test set created on the lncRNASNP dataset contains 3231 interactions involving 51 miRNAs that are entirely unseen in the training set.

When evaluating SPMLMI on this scenario, we obtained very poor results (AUROC = 0.5278, AUPRC = 0.0077), which was expected. The removal of entire nodes from the network disrupts its structural consistency, a key factor for link prediction. Indeed, the structural consistency score $\sigma_{c}$, which quantifies the network’s predictability as described in Lü *et al*. (2015), was only $\sigma_{c}=0.0028$. This indicates that, after fully removing nodes, the network loses the eigenvector patterns that underpin SPMLMI, making accurate reconstruction of missing links infeasible. While being effective when only partial edge perturbations are applied, these results highlight that SPMLMI’s performance deteriorates under drastic perturbations such as node removal: the method relies on preserved structural regularities in the network backbone to identify likely edges, and complete node removal disrupts these patterns.

In contrast, *miRInter-Trans* achieves higher AUPRC and AUROC in the “de novo” setting (Fig. 8 and Supplementary Table S4). As expected, *miRInter-Trans’*s results in the “de novo” setting are worse than in the cross-validation setting, but sufficiently accurate to support the “de novo” research for novel miRNA interactions for which no or very reduced a priori known interactions are available. Table S4 also shows that data augmentation is fundamental to boost the performance of “de novo” predictions with a marked increase in AUPRC.

**Figure 9:**
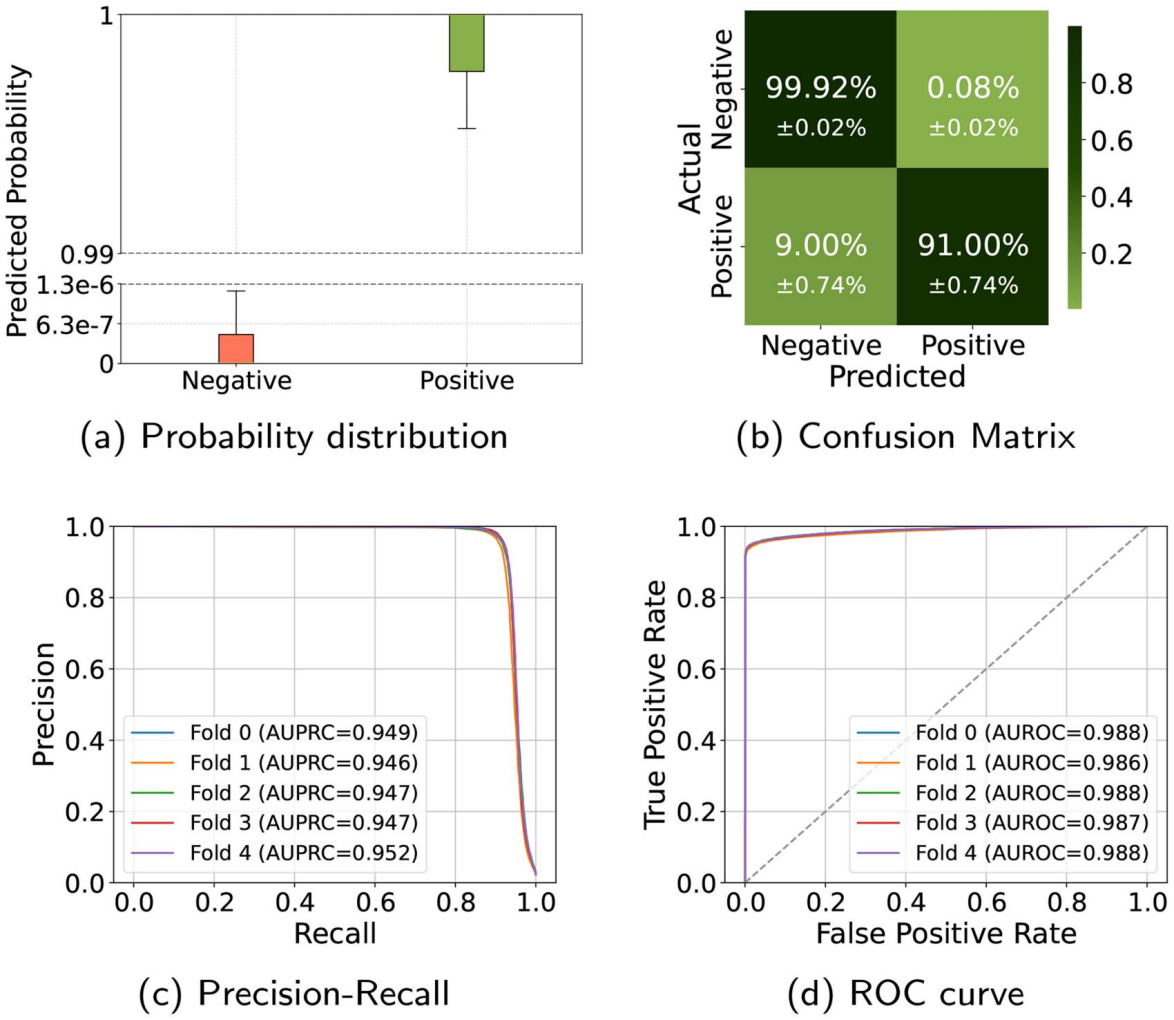
*miRInter-Trans* results using concatenated pooling on the lncRNASNP dataset (five-fold cross validation setting), using concatenated pooling. (a) Distribution of predicted probabilities for positive and negative examples. (b) Normalized confusion matrix over all the folds. (c) Precision-Recall curves on test folds. (d) ROC curves on test folds.

**Figure 10:**
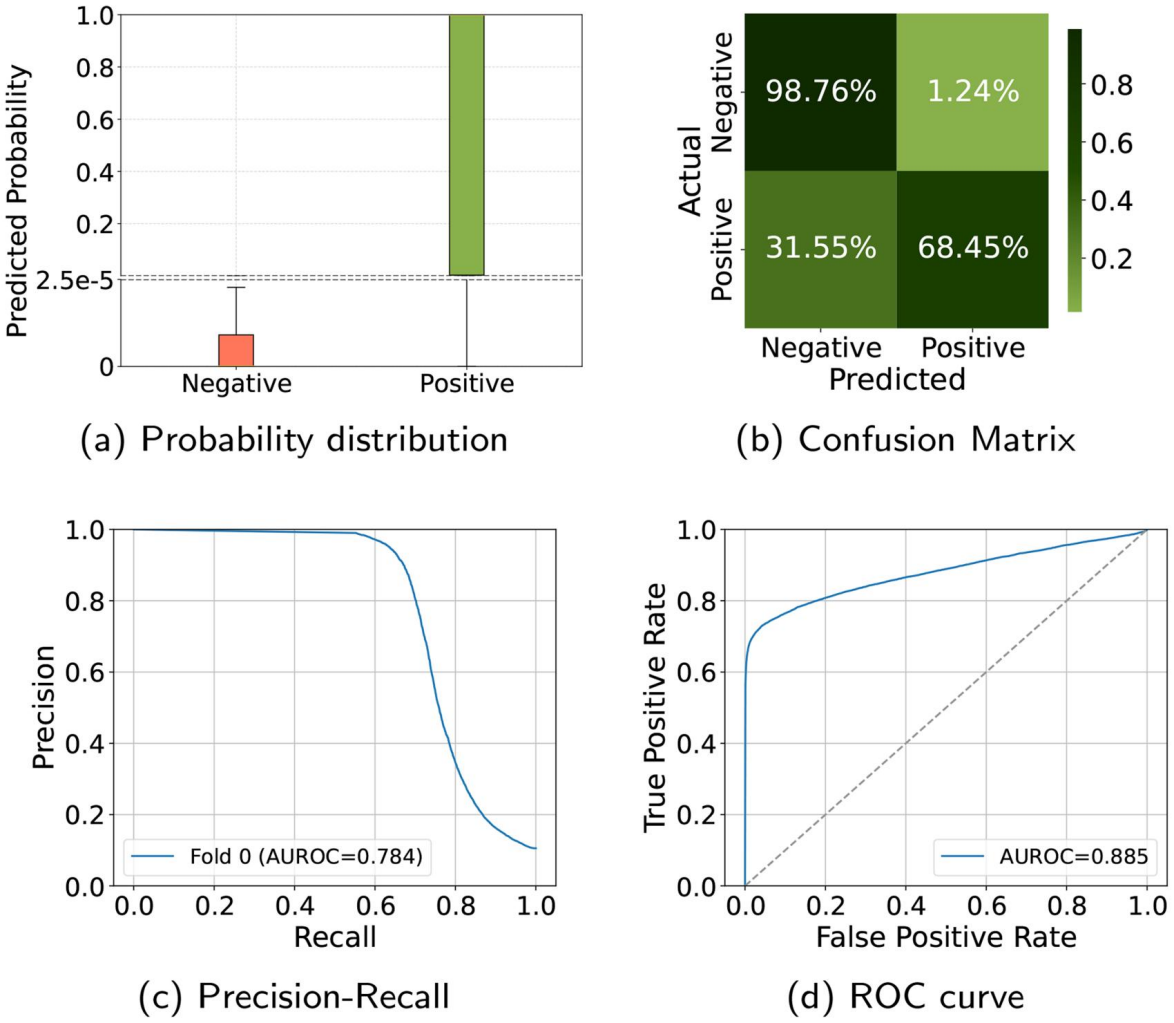
*miRInter-Trans* results on the lncRNASNP dataset (“de novo” setting), using concatenated pooling. (a) Distribution of predicted probabilities for positive and negative examples. (b) Normalized confusion matrix. (c) Precision-Recall curves on test set. (d) ROC curves on test set.

Figures 9 and 10 report *miRInter-Trans* detailed results on the lncRNASNP dataset respectively for the five-fold cross-validation and “de novo” settings. The confusion matrix in the five-fold cross-validation setting shows that less of the 10% miRNA-lncRNA interactions are missed and in practice no negative pairs are misclassified. Also the precision recall and ROC curves are very close to be ideal, indicating highly accurate ranking of RNA interactions (Fig. 9). In the most challenging “de novo” setting about 30% of miRNA-lncRNA interactions are misclassified, and also ROC and precision-recall curves suggest that there is room for further improvements (Fig. 10).

## 4. Discussion

In the following, we discuss the main factors underlying the performance and/or observed differences across datasets, interaction types, model variants and experimental settings.

We observe a consistent performance gap between lncRNASNP and RNA-KG, with markedly higher AUROC/AUPRC on lncRNASNP. This trend is consistent with the fact that the two benchmarks pose substantially different learning problems. lncRNASNP provides a considerably larger number of validated positives than RNA-KG. As a result, models trained on RNA-KG operate in a more data-limited regime. In particular, these factors tend to affect AUPRC more strongly than AUROC, since in highly imbalanced settings precision–recall performance is especially sensitive to false positives, and even a small increase in false positives can substantially reduce precision and thus AUPRC.

Nevertheless, to contextualize the RNA-KG results, we compare them with the expected performance of a random classifier (AUROC ≈0.5 and AUPRC ≈π), where $\pi =\frac{n_{\mathrm{pos}}}{n_{\mathrm{pos}}+n_{\mathrm{neg}}}$ is the prevalence of the positive (less represented) class in the evaluated split. In our setting, π≈0.05, consistent with the adopted negative-to-positive sampling ratio (i.e. $n_{\mathrm{neg}}$ negatives per positive). Under this baseline, we observe statistically significant (p<0.05) and promising performance for all three interaction types (Fig. 4). This suggests that jointly training a classifier across multiple interaction types may encourage the model to learn sequence-based patterns that generalize across interaction types and ncRNA classes, rather than relying solely on interaction-type-specific cues.

We also observed that the pooling strategy used to combine the sequence embeddings affects RNA-KG more markedly than lncRNASNP. Concatenation retains the full information from both embeddings and allows the downstream network to learn the most suitable combination of features. In contrast, average pooling can in general smooth or dilute discriminative signals leading to slightly worse performance. Finally, the max pooling strategy is the least effective pooling method, consistent with the fact that it can over-emphasize isolated high activations and amplify spurious signals, thereby harming precision in a strongly imbalanced scenario.

Moreover, the difference between training on the original versus augmented data is statistically significant on both datasets, indicating that our augmentation strategy consistently improves performance. This supports the interpretation that augmentation provides additional useful training signal, primarily acting as a robustness regularizer by exposing the model to order/orientation views of the same interactions.

From a biological standpoint, the “de novo” evaluation simulates a realistic scenario in which a newly discovered (or poorly characterized) miRNA has no experimentally validated interaction partners available, and computational predictions are needed to guide downstream investigation. In this setting, each high-scoring miRNA–ncRNA pair should be interpreted as a hypothesis that the sequence features of the miRNA (e.g. seed-related patterns and context) are compatible with regulatory recognition of the interacting ncRNA, and can therefore be used to prioritize a small subset of candidate interactions for experimental wet-lab follow-up. The observed performance decay with respect to standard cross-validation is expected, because the model cannot benefit from miRNA-specific interaction history; nevertheless, the fact that *miRInter-Trans* retains reasonable AUROC/AUPRC indicates that it captures transferable sequence-level determinants of interaction that generalize across miRNAs. Conversely, the poor performance of purely network-based link prediction under node removal highlights a biological limitation of approaches that rely on pre-existing interaction neighborhoods: when a miRNA has no known edges, sequence-driven predictors are better suited to propose initial interaction candidates. Overall, the “de novo” results support the practical use of *miRInter-Trans* as a screening tool for generating testable hypotheses on interaction partners of novel miRNAs in settings where experimental knowledge is limited.

Although the proposed methodology could be adapted to different RNA foundation models, we selected RNA-FM because it provides strong RNA-specific contextual embeddings while remaining relatively “lightweight” and practical for large-scale embedding extraction and for running the full set of ablations: RNA-FM is reported as a 12-layer model with 640-dimensional hidden size and 99M parameters. By contrast, several alternatives are one to two orders of magnitude larger (e.g. RNAGenesis with 1B parameters, AIDO.RNA (Zou *et al*. 2024) with 1.6B parameters, and BigRNA (Celaj *et al*. 2023) with 2B tunable parameters) while long-context genomic models such as Evo (7B parameters) and Evo2 (40B parameters, up to 1 Mb context) (Brixi *et al*. 2025) typically require substantially higher memory/compute and would significantly expand the computational footprint and the scope of the present study.

## 5. Conclusions

Our results demonstrate that transformer-based sequence embeddings can effectively be used predict miRNA interactions directly from sequence, without relying on structural information or thermodynamic parameters.

Augmentation strategies also play a key role in improving performance, since enlarging the available training data can consistently boost the generalization capabilities of the model.

The results also show that our transformer-based models can be successfully applied even to the challenging “de novo” prediction of miRNA interactions, a task that naturally arises from experimental situations characterized by no or very limited interaction data available for newly discovered miRNAs.

We obtained the best results by training from scratch the prediction neural network using the embedded representations of the RNA sequences generated by RNA-FM. We tried also to fine-tune the RNA-FM embeddings with the RNA-KG data, but we did not achieve improved results, since it is likely that most of the miRNA and lncRNA in RNA-KG have been already used to train the RNA-FM transformer.

These promising results on RNA-KG, especially when contrasted with a random-classifier baseline, suggest that our model may learn sequence-based interaction signals that generalize across different ncRNA classes and interaction types. Our ongoing work aims to investigate this aspect more systematically (Nicolini *et al*. 2026).

Despite these successful results, this work presents several limitations, that could constitute the object of future research.

At first, *miRInter-Trans* has been trained to three types of miRNA interactions: on the one hand this enlarges the scope of miRNA interactions with respect to previous works (Kang *et al*. 2020, Yang *et al*. 2025, Li *et al*. 2025), but on the other hand we could consider a larger set of ncRNA interactions, including e.g. other types of ncRNA interactions available from public resources such as RNA-KG or RNAInter (Kang *et al*. 2022). Moreover the maximum length of the ncRNA allowed by *miRInter-Trans* is limited to 1022, due to the internal limitations of the underlying RNA-FM model. In this work we intentionally fixed RNA-FM as the embedding backbone to keep the experimental analysis controlled while systematically evaluating downstream choices (e.g. pooling/combination strategies and data augmentation techniques); nonetheless, replacing RNA-FM with larger and/or long-context RNA foundation models (e.g. AIDO.RNA (Zou *et al*. 2024), BigRNA (Celaj *et al*. 2023), RNAGenesis (Zhang *et al*. 2024), Evo/Evo2 (Nguyen *et al*. 2024, Brixi *et al*. 2025), GenerRNA (Zhao *et al*. 2024), or RNAErnie (Wang *et al*. 2024)) is a natural direction for future work to potentially improve accuracy and extend coverage to longer transcripts.

A second limitation of this work is that we kept our interaction module relatively simple compared to the richness of the transformer embeddings. Indeed *miRInter-Trans* adopts classical pooling strategies (e.g. maximum or average) to obtain fixed-length sequence representations. Nevertheless, future work could investigate more expressive interaction and pooling mechanisms, such as attention-based pooling (including self-attention pooling) and cross-attention between RNA sequences, as well as other advanced pooling techniques recently proposed in the context of protein modeling (Hoang and Singh 2025), to better exploit contextual information, learn interaction-specific alignment and importance patterns across sequences, and potentially improve performance.

Another limitation of this work is the lack of a systematic interpretation of interaction results, and in perspective we plan to explore explainable techniques that can help highlight sequence regions that contribute to predicted interactions.

Summarizing, this work proposes a novel deep-learning based model for the computational prediction of different types of miRNA interactions leveraging specialized neural networks trained on top of a RNA transformer and suggests new research lines for the design of deep learning models for the prediction of ncRNA interactions.

## Supplementary Material

vbag073_Supplementary_Data

## Data Availability

The Data and Code underlying this article are available in GitHub at: https://github.com/AnacletoLAB/miRInter-Trans. All datasets used in this study are derived from publicly available sources as referenced in the manuscript.

## Footnotes

1 We used version 2.0 of lncRNASNP, available at http://bioinfo.life.hust.edu.cn/lncRNASNP2.

2 SPMLMI code and data: https://github.com/xumingmin/SPMLMI.

3 We generated the five cross-validation folds using the authors’ code available at: https://github.com/xumingmin/SPMLMI.
