# Supplementary material for: MiRInter-Trans: a transformer-based framework for microRNA interaction prediction
Source: Bioinform Adv. 2026 Mar 9;6(1):vbag073. doi: 10.1093/bioadv/vbag073 (PMC13268801; doi:10.1093/bioadv/vbag073)
Supplement: vbag073_Supplementary_Data [file vbag073_supplementary_data.zip › miRInter_Trans_final_submission_supplementary.pdf]

MiRInter-Trans: a Transformer-Based Framework for microRNA Interaction Prediction – Supplementary Information

Marco Nicolini, Federico Stacchietti, Francisco Javier Molina, Carlos Cano, Jesus Alcala-Fdez, Alberto Paccanaro, Elena Casiraghi and Giorgio Valentini

Figures

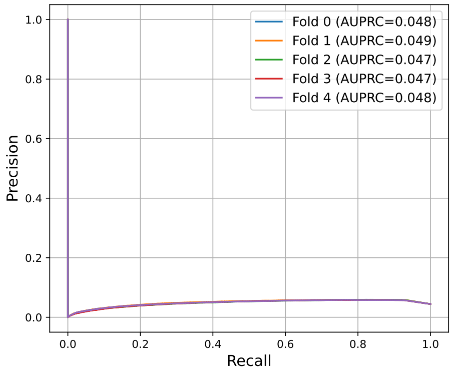

(a)

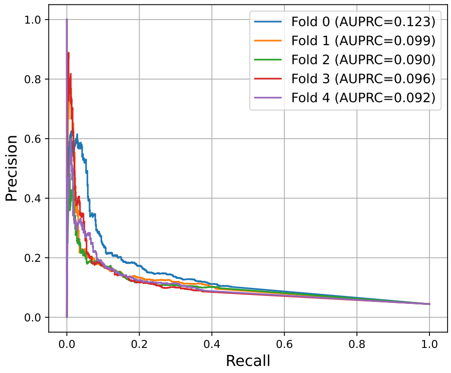

(b)

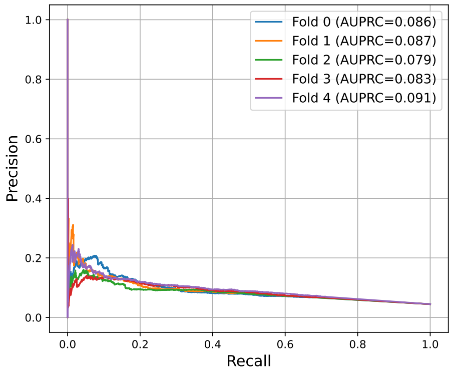

(c)

Figure S1 IntaRNA results on RNA-KG data: first row miRNA-lncRNA, second miRNA-miRNA and third miRNA-snoRNA interaction prediction. (a, b, c) Precision-Recall curves on test folds.

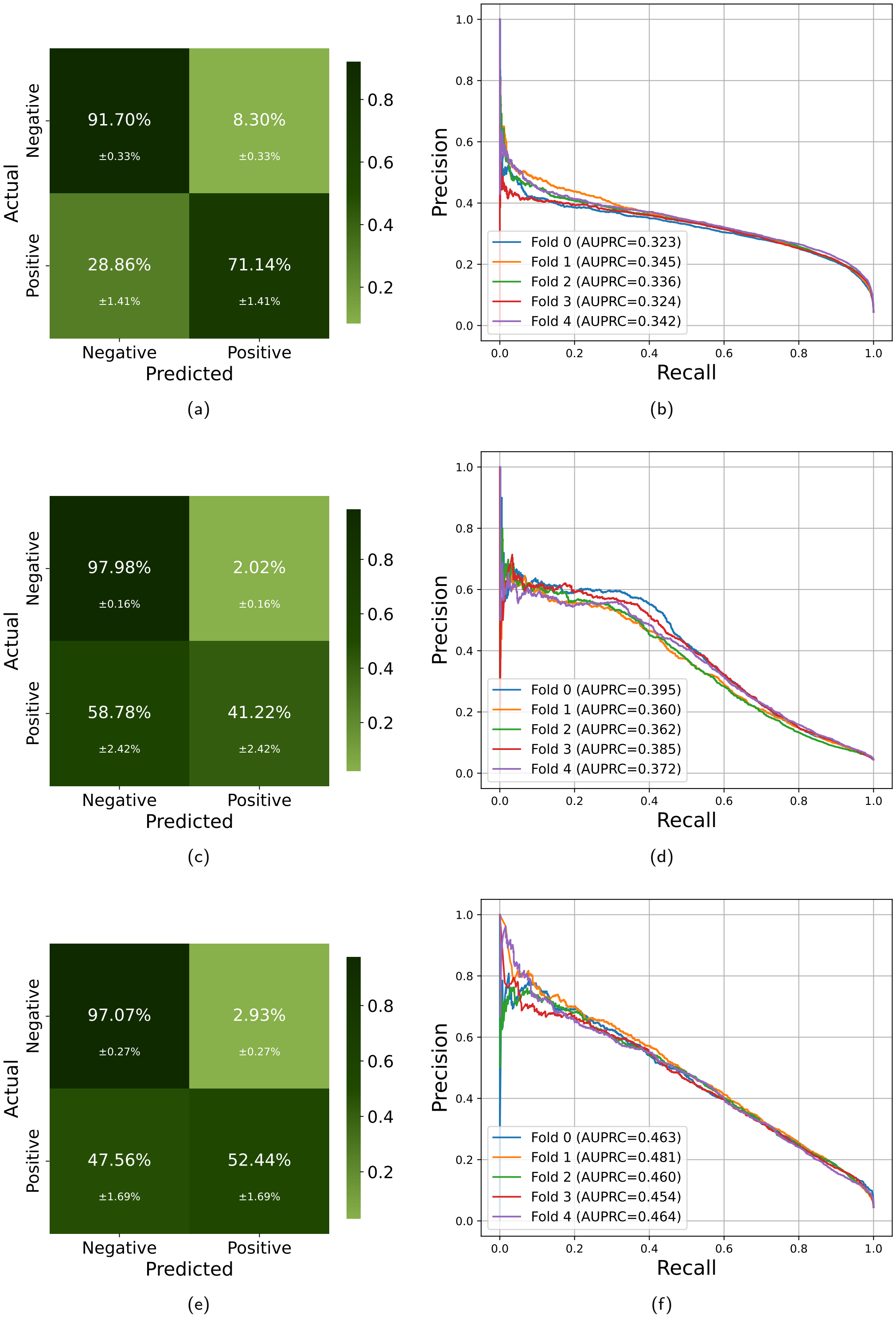

Figure S2 *miRInter-Trans* results on RNA-KG data: first row miRNA-lncRNA, second miRNA-miRNA and third miRNA-snoRNA interaction prediction. (a,c,e) Normalized confusion matrix of the classifier (averaged across folds) (b,d,f) Precision-Recall curves on test folds.

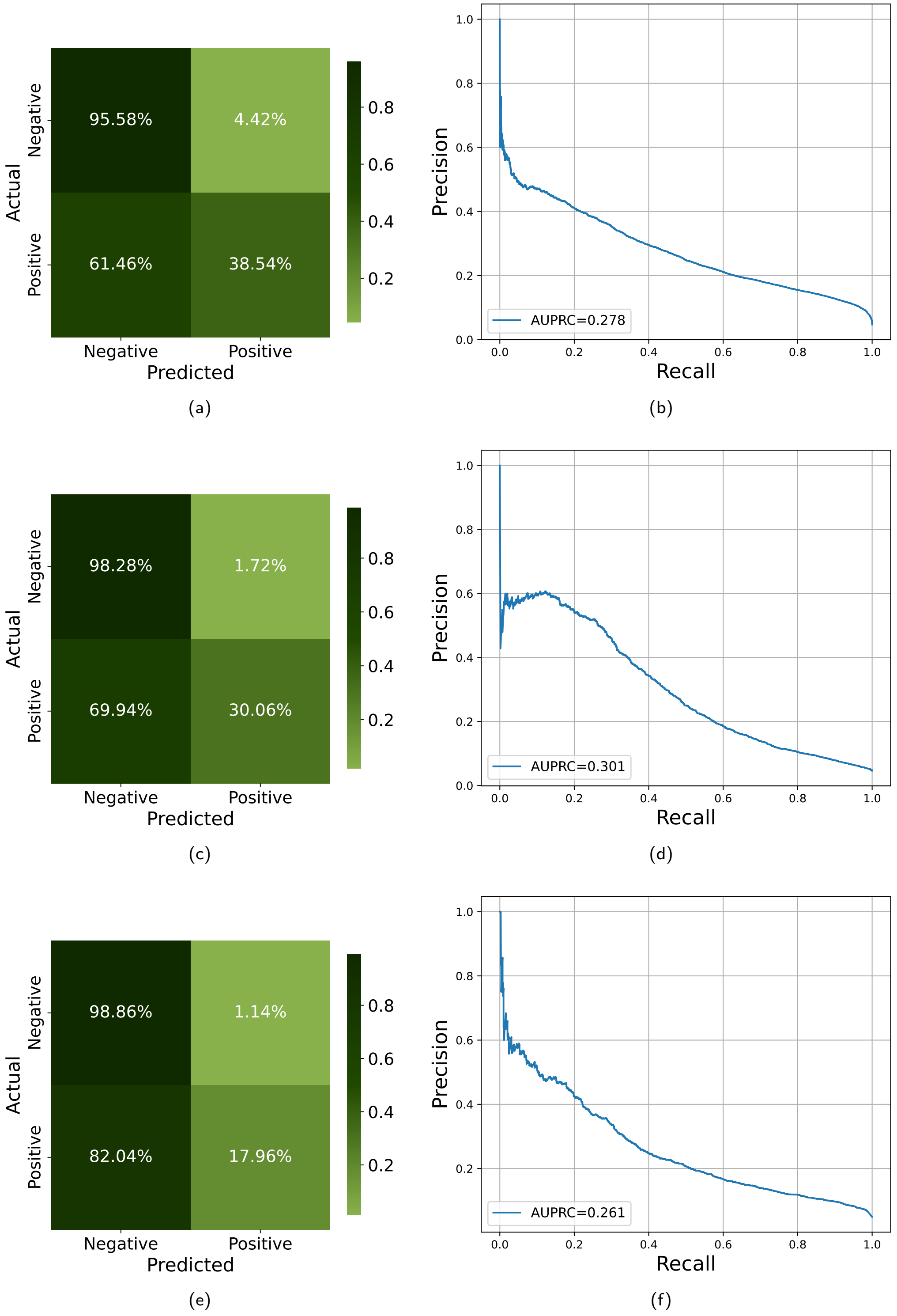

Figure S3 *miRInter-Trans* results on RNA-KG data for de-novo miRNA interaction prediction: first row miRNA-lncRNA, second miRNA-miRNA and third miRNA-snoRNA interaction prediction. (a,c,e) Normalized confusion matrix of the classifier (b,d,f) Precision-Recall curves on test set.

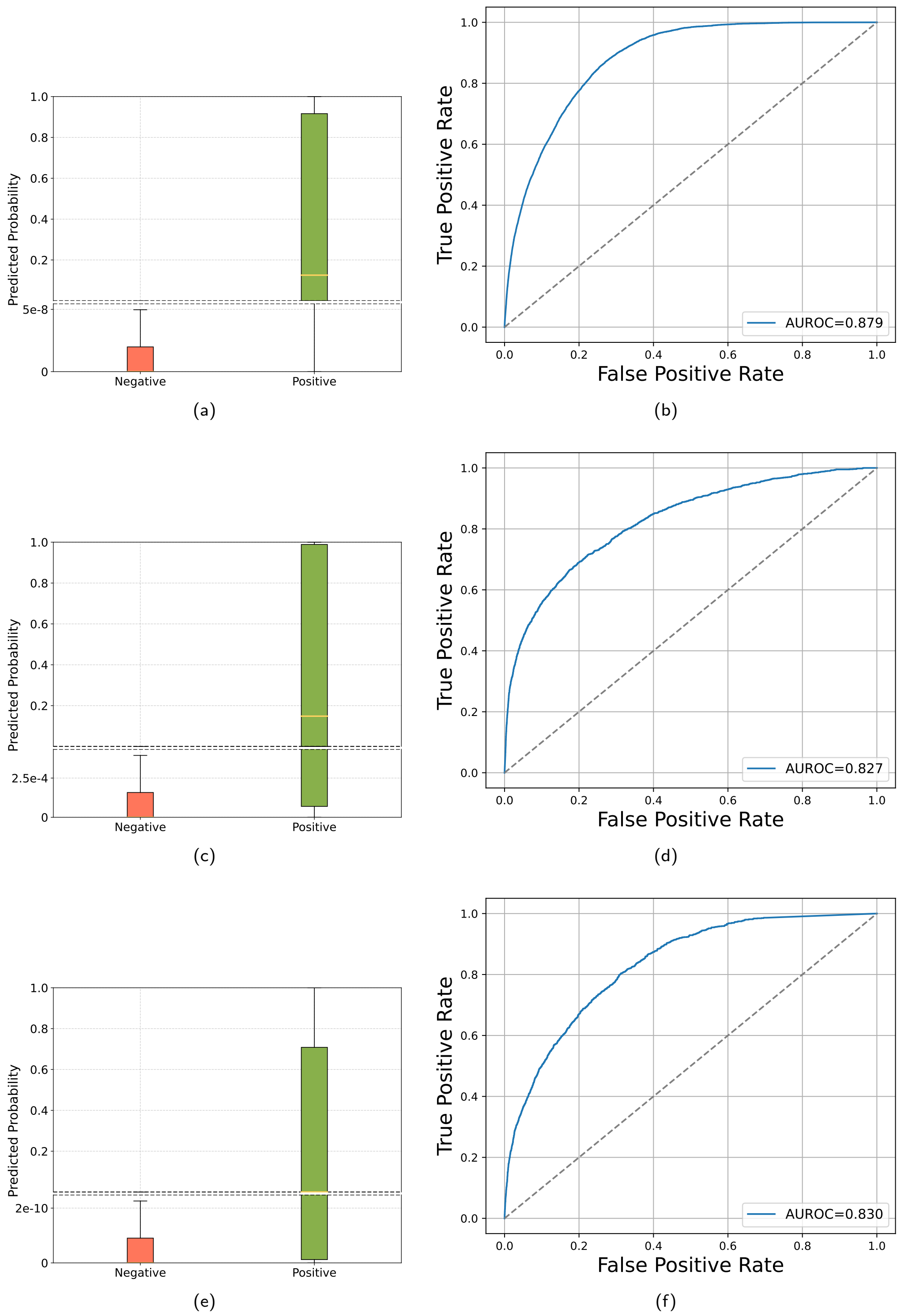

Figure S4 *miRInter-Trans* results on RNA-KG data for de-novo miRNA interaction predction: first row miRNA-lncRNA, second miRNA-miRNA and third miRNA-snoRNA interaction prediction. (a,c,e) Distribution of predicted probabilities for positive and negative examples. (b,d,f) ROC curve on test set.

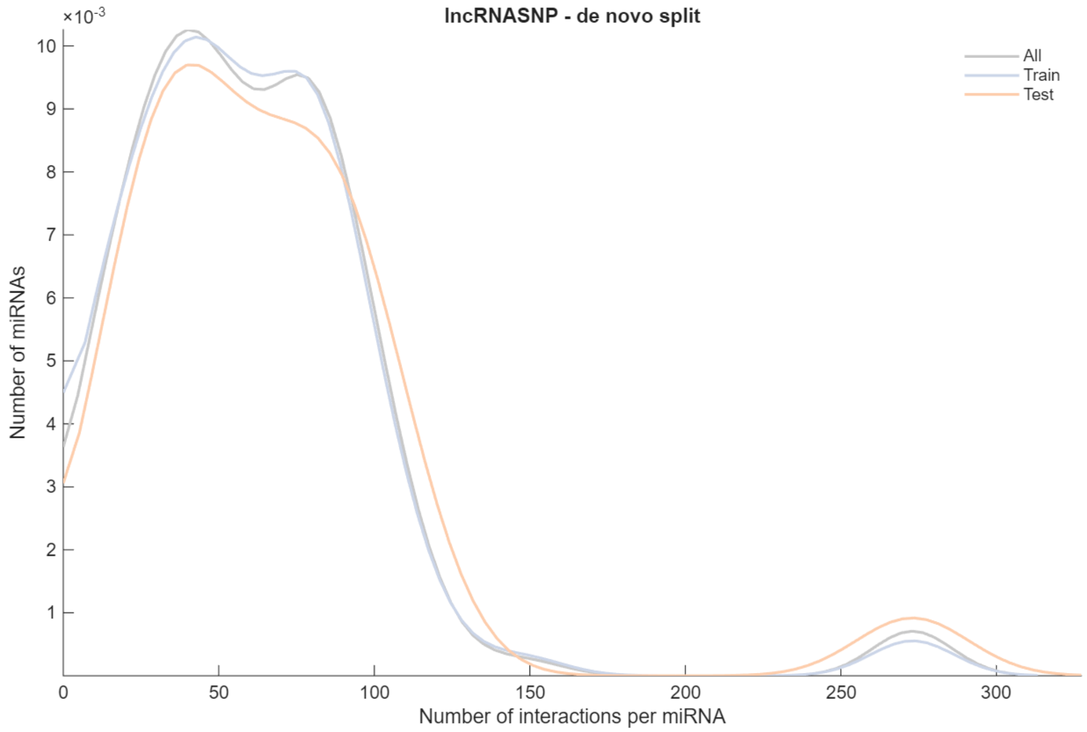

Figure S5 "De novo" experimental setting with lncRNASNP data. Normalized histogram of the frequency of miRNA interactions in the training, and test sets and in the overall data.

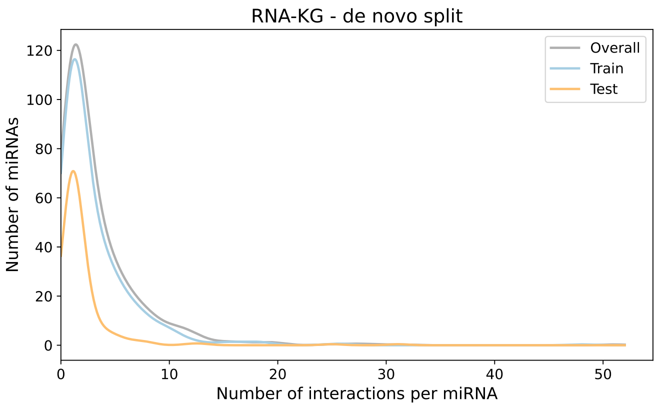

(a) miRNA-miRNA

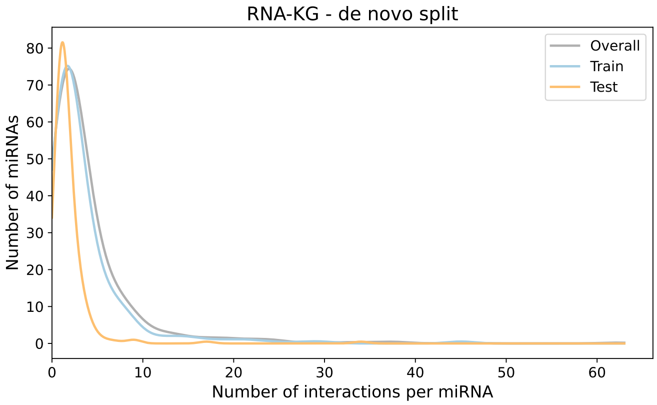

(b) miRNA-snoRNA

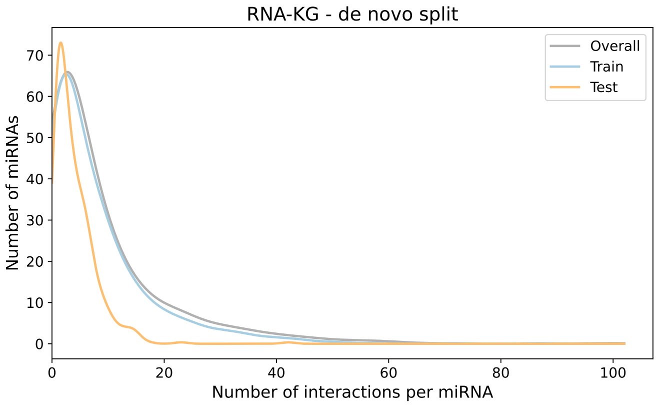

(c) miRNA-lncRNA

Figure S6 "De novo" exeprimental setting with RNA-KG data. Normalized histogram of the frequency of miRNA interactions in the overall, training, and test set. (a) miRNA-miRNA; (b) miRNA-snoRNA (c) miRNA-lncRNA.

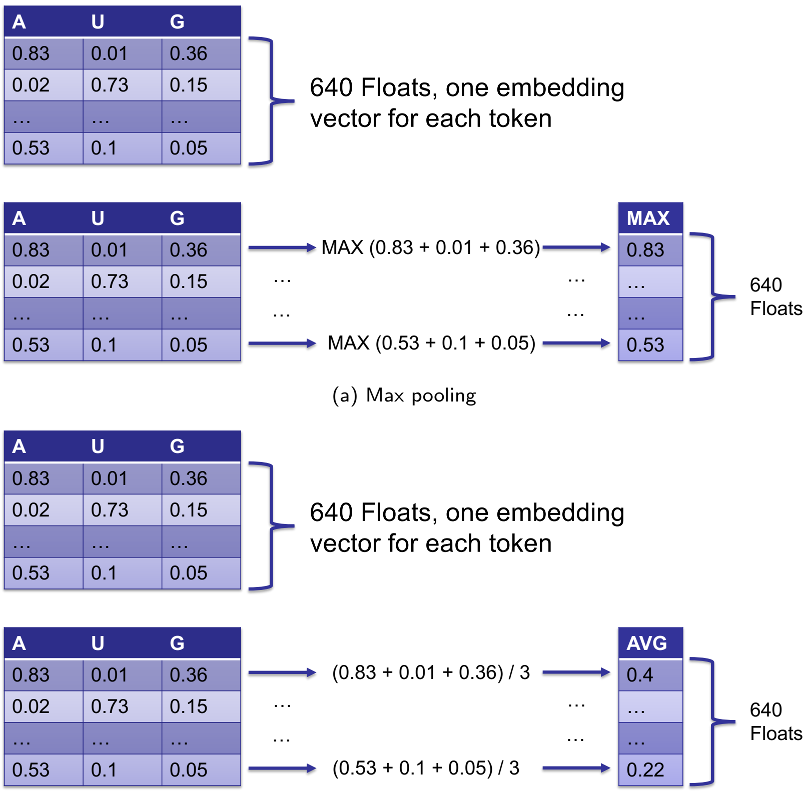

(a) Max pooling

(b) Average pooling

Figure S7 Illustration of average and max pooling applied to a toy RNA sequence of length three. Each nucleotide is mapped to a token embedding, forming an embedding matrix of size *sequence length* $\times$ *embedding dimension*. Pooling reduces this matrix to a fixed-size vector representation.

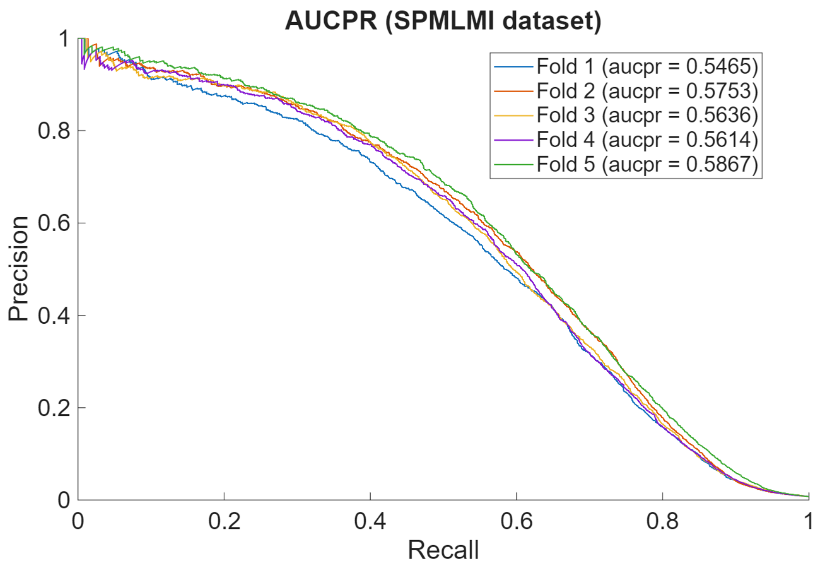

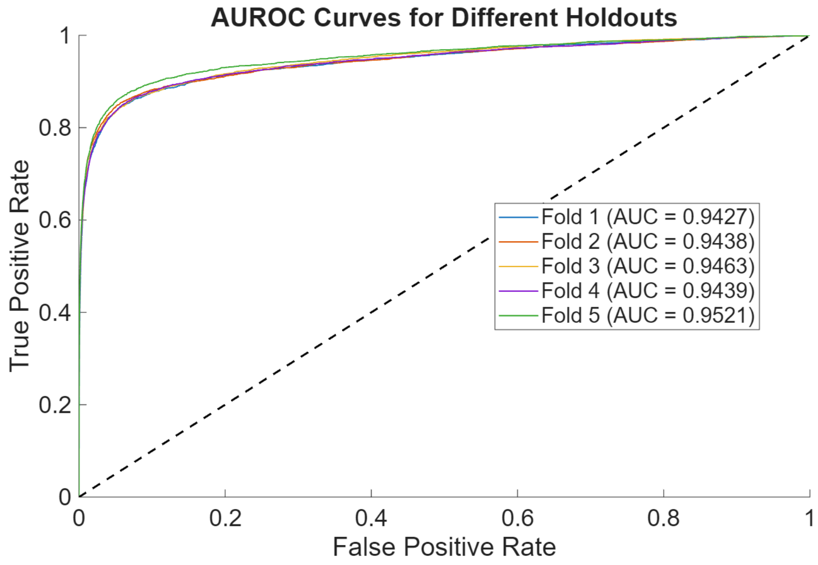

Figure S8 AUPRC (left) and AUROC (right) results achieved by SPMLMI across the five folds on the lncRNASNP dataset.

Tables

Table S1. Performance (mean $\pm$ σ) of miRInter-Trans with the three pooling strategies on the RNA-KG dataset, where σ denotes the *sample* standard deviation across the five folds. Reported p-values correspond to one-sided paired t-tests (the best strategy - miRInter-Trans-Concat - with the remaining methods) for AUROC and AUPRC across the three interaction types. Significant p-values ($p < 0.05$) are in bold.

Interaction Type	Method	AUROC (mean $\pm$ σ)	AUPRC (mean $\pm$ σ)	p_{AUROC}	p_{AUPRC}
miRNA–lncRNA	miRInter-Trans-Concat	**0.932 $\pm$ 0.002**	**0.334 $\pm$ 0.010**	-	-
	miRInter-Trans-AVG	0.927 $\pm$ 0.001	0.311 $\pm$ 0.004	**0.00094**	**0.00246**
	miRInter-Trans-MAX	0.931 $\pm$ 0.001	0.331 $\pm$ 0.003	0.11384	0.24256
	miRInter-Trans-Concat-noAUG	0.8887 $\pm$ 0.0035	0.2383 $\pm$ 0.0046	-	-
	miRInter-Trans-AVG-noAUG	0.8828 $\pm$ 0.002	0.2212 $\pm$ 0.0052	-	-
	miRInter-Trans-MAX-noAUG	0.8867 $\pm$ 0.0034	0.2338 $\pm$ 0.0094	-	-
miRNA-miRNA	miRInter-Trans-Concat	**0.878 $\pm$ 0.007**	**0.375 $\pm$ 0.015**	-	-
	miRInter-Trans-AVG	0.870 $\pm$ 0.004	0.354 $\pm$ 0.012	**0.01172**	**0.01125**
	miRInter-Trans-MAX	0.871 $\pm$ 0.009	0.364 $\pm$ 0.022	**0.01835**	0.05076
	miRInter-Trans-Concat-noAUG	0.8659 $\pm$ 0.0103	0.3289 $\pm$ 0.0320	-	-
	miRInter-Trans-AVG-noAUG	0.8566 $\pm$ 0.01	0.3339 $\pm$ 0.0119	-	-
	miRInter-Trans-MAX-noAUG	0.8627 $\pm$ 0.0056	0.3315 $\pm$ 0.0233	-	-
miRNA-snoRNA	miRInter-Trans-Concat	**0.931 $\pm$ 0.002**	**0.465 $\pm$ 0.010**	-	-
	miRInter-Trans-AVG	0.926 $\pm$ 0.003	0.445 $\pm$ 0.008	**0.00066**	**0.00727**
	miRInter-Trans-MAX	0.927 $\pm$ 0.003	0.450 $\pm$ 0.004	**0.00067**	**0.02467**
	miRInter-Trans-Concat-noAUG	0.909 $\pm$ 0.0057	0.3744 $\pm$ 0.0282	-	-
	miRInter-Trans-AVG-noAUG	0.9004 $\pm$ 0.0018	0.3711 $\pm$ 0.0188	-	-
	miRInter-Trans-MAX-noAUG	0.9065 $\pm$ 0.0092	0.3673 $\pm$ 0.0343	-	-

Table S2. Performance (mean $\pm$ σ) of miRInter-Trans-Concat compared with IntaRNA and Random baseline for AUROC and AUPRC on the RNA-KG dataset, where σ denotes the *sample* standard deviation across the five folds. Reported p-values correspond to one-sided paired t-tests (miRInter-Trans-Concat with the remaining methods). Significant p-values ($p < 0.05$) are in bold.

Interaction Type	Method	AUROC (mean $\pm$ σ)	AUPRC (mean $\pm$ σ)	p_{AUROC}	p_{AUPRC}
miRNA–lncRNA	miRInter-Trans-Concat	**0.933 $\pm$ 0.002**	**0.334 $\pm$ 0.010**	–	–
	IntaRNA	0.582 $\pm$ 0.005	0.048 $\pm$ 0.001	**7.9E-9**	**1.9E-7**
	Random	0.500 $\pm$ 0.000	0.047 $\pm$ 0.000	**1.0E-10**	**2.1E-7**
miRNA–miRNA	miRInter-Trans-Concat	**0.878 $\pm$ 0.007**	**0.375 $\pm$ 0.015**	–	–
	IntaRNA	0.622 $\pm$ 0.016	0.100 $\pm$ 0.013	**4.3E-6**	**2.4E-7**
	Random	0.500 $\pm$ 0.000	0.047 $\pm$ 0.000	**1.4E-8**	**5.3E-7**
miRNA–snoRNA	miRInter-Trans-Concat	**0.931 $\pm$ 0.002**	**0.465 $\pm$ 0.010**	–	–
	IntaRNA	0.656 $\pm$ 0.009	0.085 $\pm$ 0.004	**3.2E-7**	**4.4E-8**
	Random	0.500 $\pm$ 0.000	0.047 $\pm$ 0.000	**8.1E-11**	**4.1E-8**

Table S3. Performance in the *de novo* experimental setting on the RNA-KG dataset for the three pooling strategies of *miRInter-Trans*. Values are single-test results (no standard deviation). Best value per interaction and metric is in bold.

Interaction Type	Method	AUROC	AUPRC
miRNA–lncRNA	miRInter-Trans-Concat	**0.879**	0.278
	miRInter-Trans-AVG	0.878	**0.282**
	miRInter-Trans-MAX	0.869	0.251
miRNA–miRNA	miRInter-Trans-Concat	0.826	0.301
	miRInter-Trans-AVG	**0.835**	**0.320**
	miRInter-Trans-MAX	0.816	0.291
miRNA–snoRNA	miRInter-Trans-Concat	0.830	0.261
	miRInter-Trans-AVG	**0.850**	**0.283**
	miRInter-Trans-MAX	0.772	0.232

Table S4. Performance on the lncRNASNP data set with the 5-fold cross-validation and "de novo" experimental settings. In the 5-fold cross-validation setting, values are mean performance across folds ± standard deviation; the suffix noAUG refers to an experimental setting with no augmented data.

Exp. setting	Approach	AUPRC(sd) / AUROC(sd)
5-fold CV	miRInter-Trans-concat	**0.948** ± 0.001 / **0.987** ± 0.002
	miRInter-Trans-AVG	0.941 ± 0.003 / 0.985 ± 0.003
	miRInter-Trans-MAX	0.945 ± 0.003 / 0.987 ± 0.001
	miRInter-Trans-concat-noAUG	0.894 ± 0.006 / 0.976 ± 0.001
	miRInter-Trans-AVG-noAUG	0.875 ± 0.008 / 0.972 ± 0.003
	miRInter-Trans-MAX-noAUG	0.901 ± 0.006 / 0.977 ± 0.001
"de novo" prediction	miRInter-Trans-concat	**0.784** / **0.885**
	miRInter-Trans-AVG	0.778 / 0.875
	miRInter-Trans-MAX	0.775 / 0.879
	miRInter-Trans-concat-noAUG	0.431 / 0.764
	miRInter-Trans-AVG-noAUG	0.442 / 0.765
	miRInter-Trans-MAX-noAUG	0.438 / 0.743

Table S5. Performance (mean ± σ) of miRInter-Trans with the three pooling strategies on the lncRNASNP dataset, where σ denotes the *sample* standard deviation across the five folds. Reported p-values correspond to one-sided paired t-tests (miRInter-Trans-Concat vs the remaining poolings). Significant p-values ($p < 0.05$) are in bold.

Interaction Type	Method	AUROC (mean ± σ)	AUPRC (mean ± σ)	p_{AUROC}	p_{AUPRC}
miRNA–lncRNA	miRInter-Trans-Concat	**0.987 ± 0.001**	**0.948 ± 0.002**	–	–
	miRInter-Trans-AVG	0.985 ± 0.001	0.942 ± 0.003	**9.5E-4**	**8.04E-3**
	miRInter-Trans-MAX	0.987 ± 0.001	0.945 ± 0.004	0.217	**2.33E-2**

Table S6. Performance (mean ± σ) of miRInter-Trans-Concat compared with SPMLMI and Random baseline on the lncRNASNP dataset, where σ denotes the *sample* standard deviation across the five folds. Reported p-values correspond to one-sided paired t-tests (miRInter-Trans-Concat vs the other methods). Significant p-values ($p < 0.05$) are in bold.

Interaction Type	Method	AUROC (mean ± σ)	AUPRC (mean ± σ)	p_{AUROC}	p_{AUPRC}
miRNA–lncRNA	miRInter-Trans-Concat	**0.987 ± 0.001**	**0.948 ± 0.002**	–	–
	SPMLMI	0.946 ± 0.004	0.567 ± 0.015	**7.09E-6**	**2.48E-7**
	Random	0.500 ± 0.000	0.037 ± 0.000	**5.03E-13**	**6.70E-12**
