# Supplementary material for: MiRInter-Trans: a transformer-based framework for microRNA interaction prediction
Source: Bioinform Adv. 2026 Mar 9;6(1):vbag073. doi: 10.1093/bioadv/vbag073 (PMC13268801; doi:10.1093/bioadv/vbag073)
Supplement: vbag073_Supplementary_Data [file vbag073_supplementary_data.zip › miRInter_Trans_submission_supplementary.pdf.pdf]

# MiRInter-Trans: a Transformer-Based Framework for microRNA Interaction Prediction – Supplementary Information

Marco Nicolini, Federico Stacchietti, Francisco Javier Molina, Carlos Cano, Jesus Alcala-Fdez, Iago S. D. Riveiro, Alberto Paccanaro, Elena Casiraghi and Giorgio Valentini

# Figures

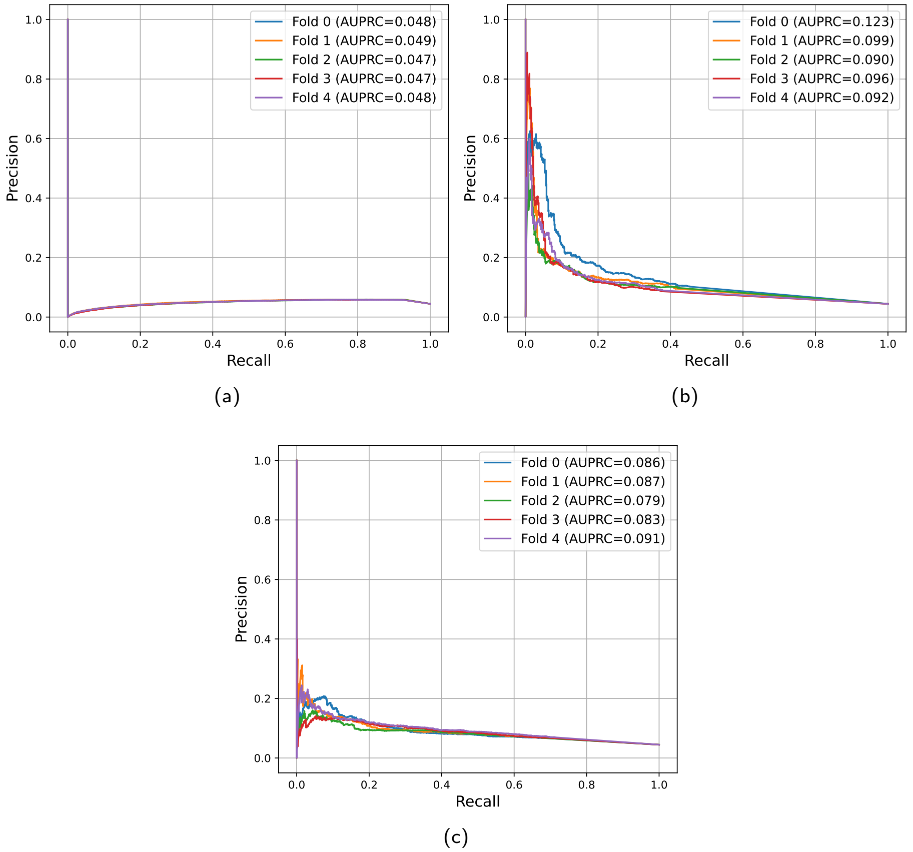


**Figure S1** IntaRNA results on RNA-KG data: first row miRNA-lncRNA, second miRNA-miRNA and third miRNA-snoRNA interaction prediction. (a, b, c) Precision-Recall curves on test folds.

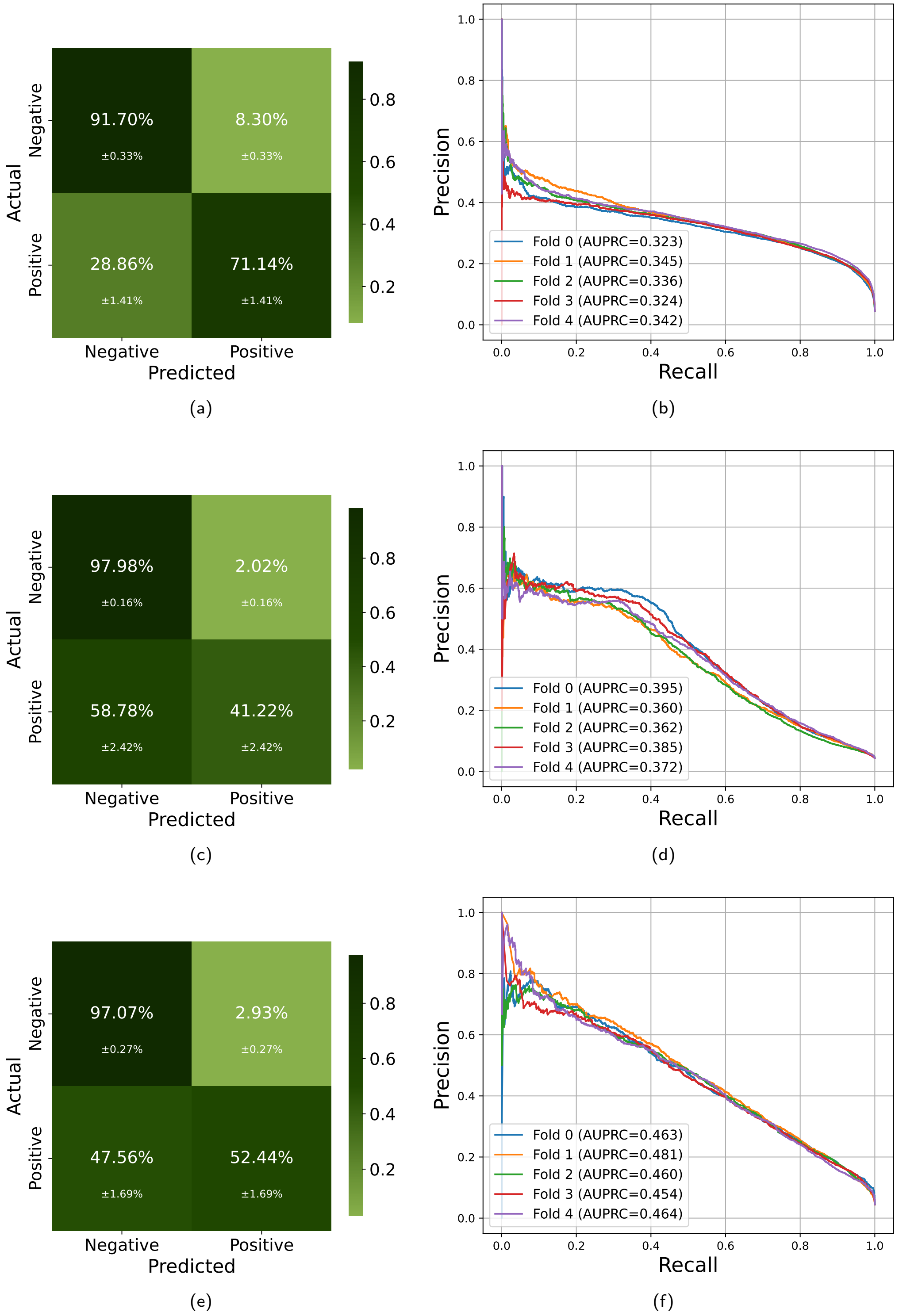


**Figure S2** *miRInter-Trans* results on RNA-KG data: first row miRNA-lncRNA, second miRNA-miRNA and third miRNA-snoRNA interaction prediction. (a,c,e) Normalized confusion matrix of the classifier (averaged across folds) (b,d,f) Precision-Recall curves on test folds.

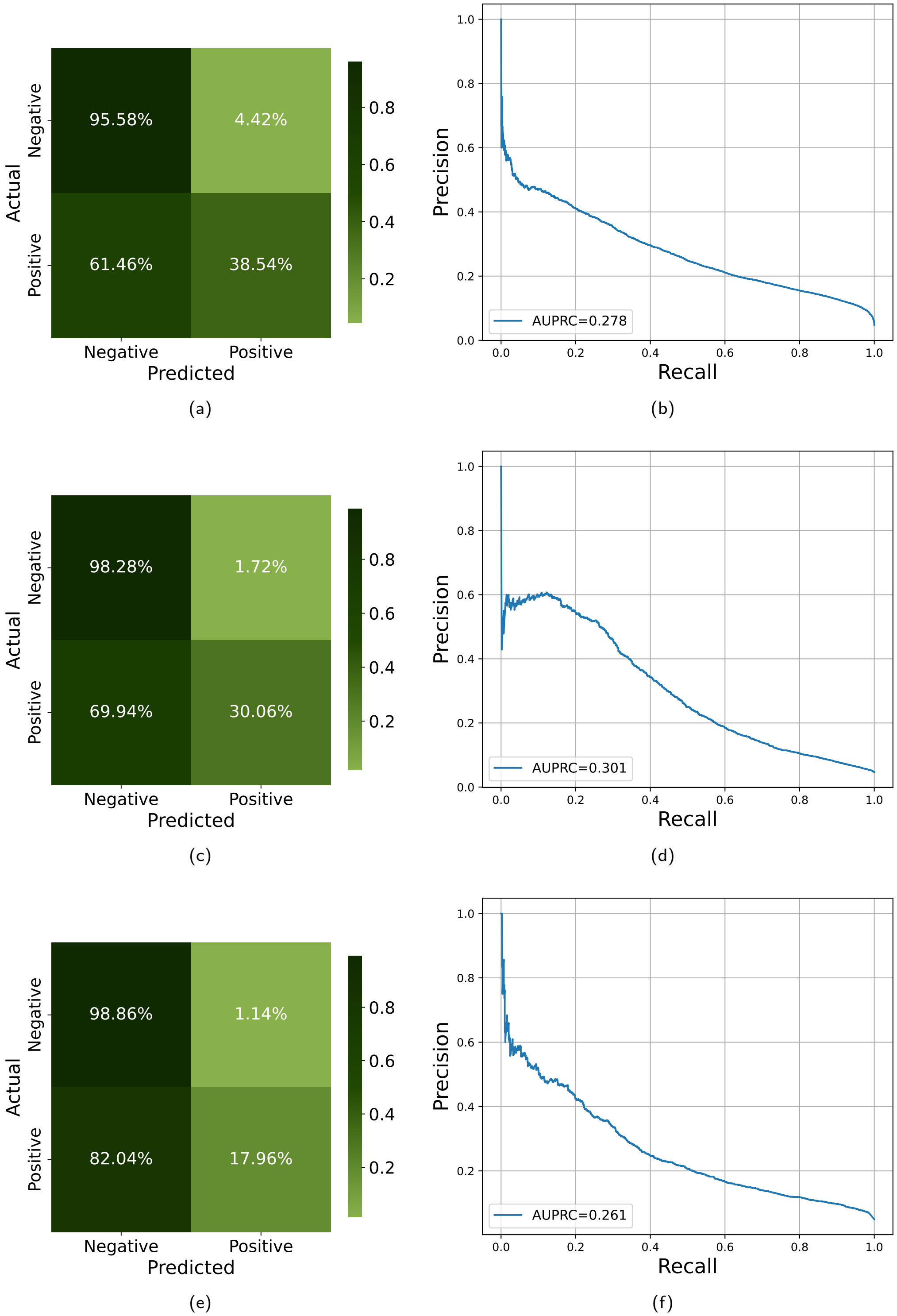


**Figure S3** *miRInter-Trans* results on RNA-KG data for de-novo miRNA interaction prediction: first row miRNA-lncRNA, second miRNA-miRNA and third miRNA-snoRNA interaction prediction. (a,c,e) Normalized confusion matrix of the classifier (b,d,f) Precision-Recall curves on test set.

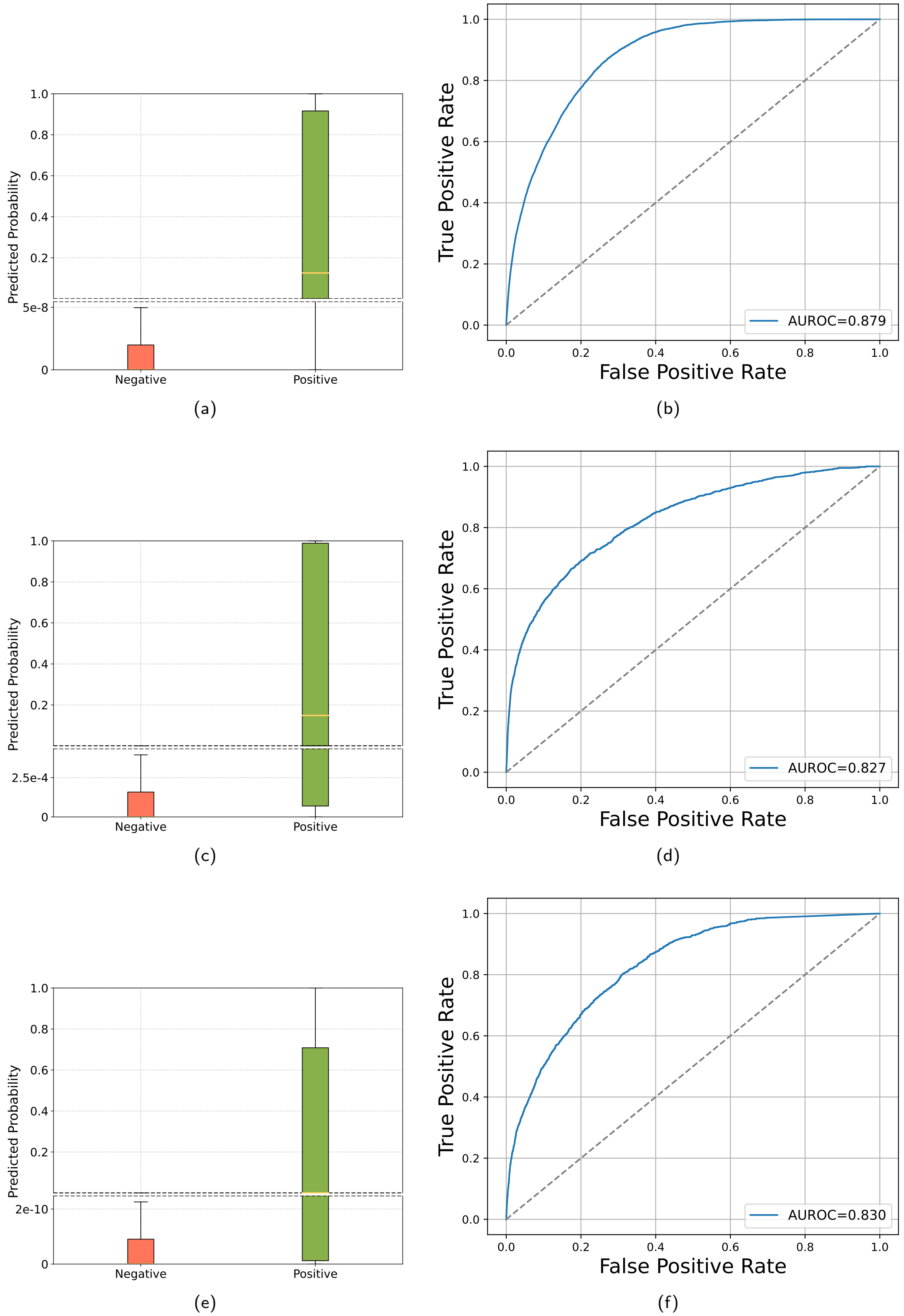


**Figure S4** *miRInter-Trans* results on RNA-KG data for de-novo miRNA interaction predction: first row miRNA-lncRNA, second miRNA-miRNA and third miRNA-snoRNA interaction prediction. (a,c,e) Distribution of predicted probabilities for positive and negative examples. (b,d,f) ROC curve on test set.

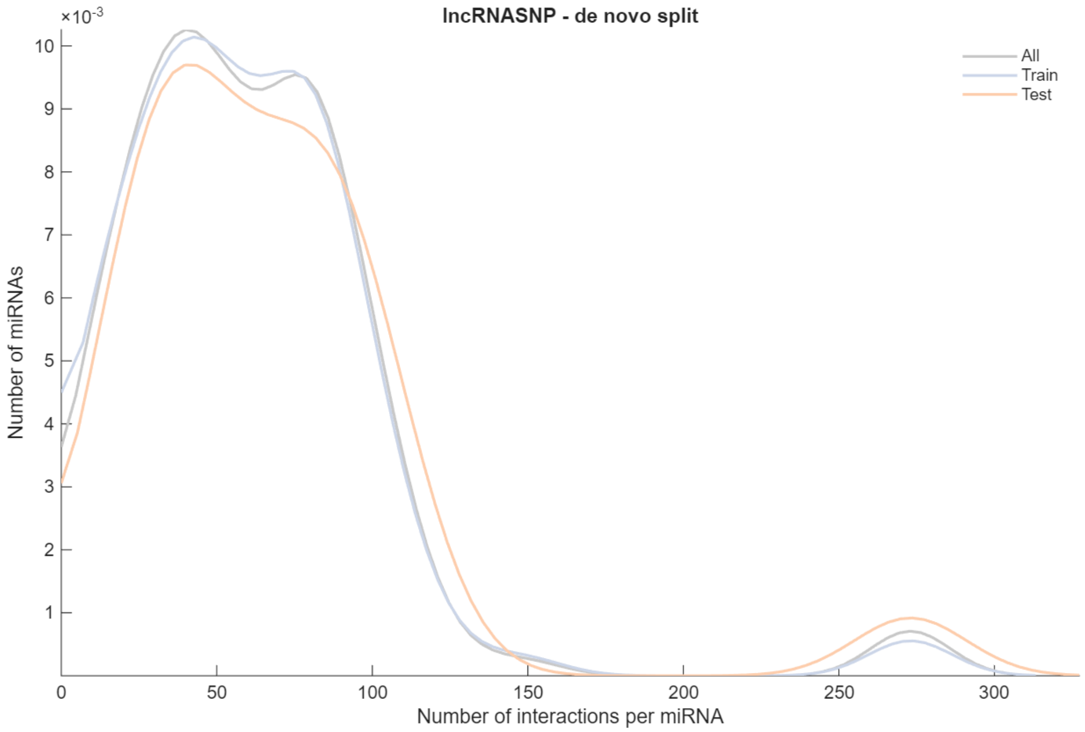


**Figure S5** "De novo" experimental setting with lncRNASNP data. Normalized histogram of the frequency of miRNA interactions in the training, and test sets and in the overall data.

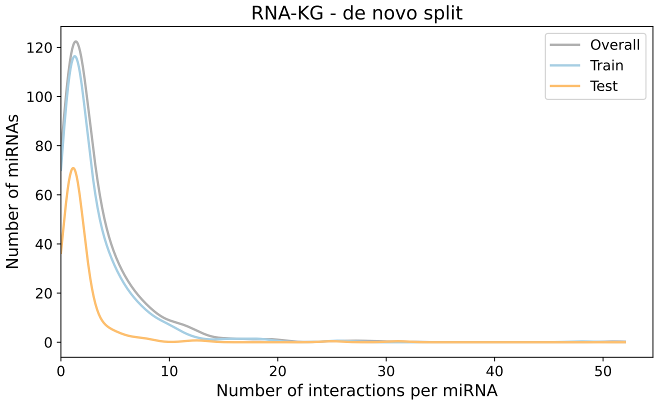


(a) miRNA-miRNA

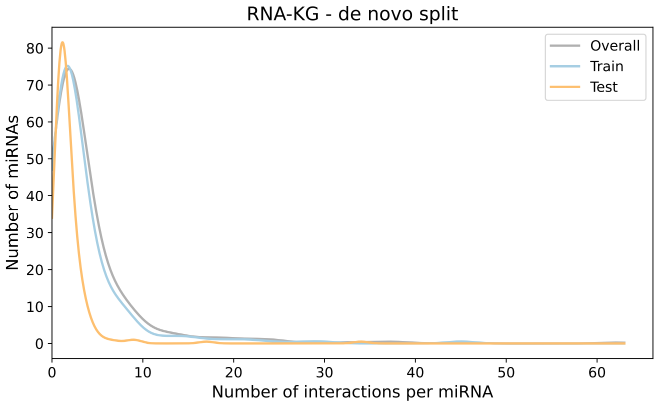


(b) miRNA-snoRNA

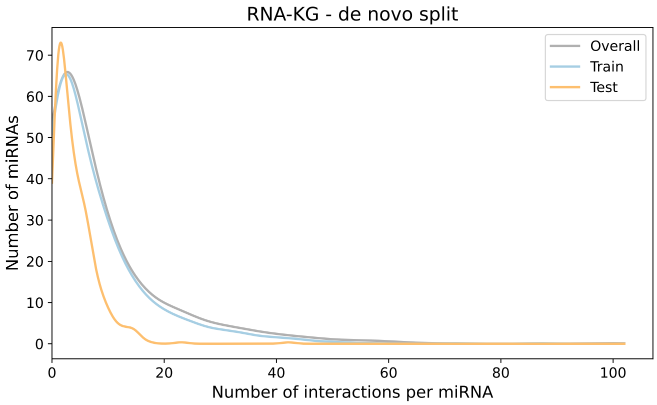


(c) miRNA-lncRNA

**Figure S6** "De novo" exeprimental setting with RNA-KG data. Normalized histogram of the frequency of miRNA interactions in the overall, training, and test set. (a) miRNA-miRNA; (b) miRNA-snoRNA (c) miRNA-lncRNA.

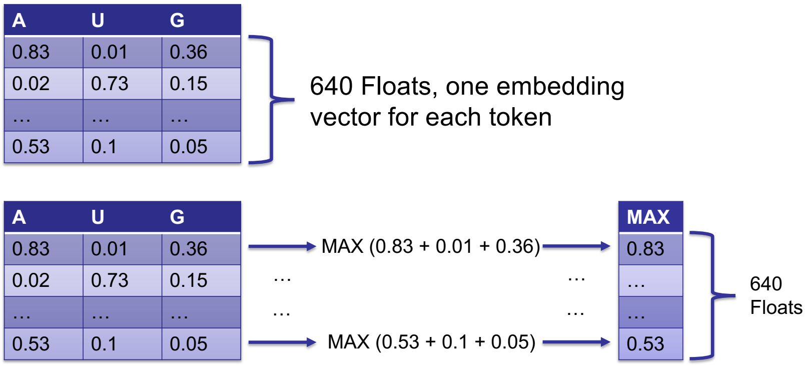


(a) Max pooling

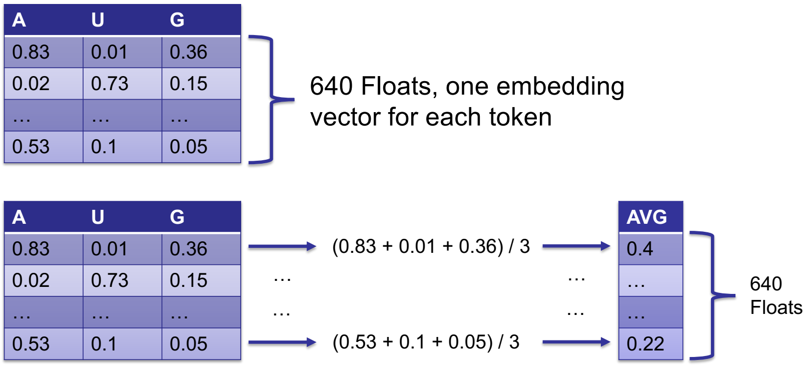


(b) Average pooling

**Figure S7** Illustration of average and max pooling applied to a toy RNA sequence of length three. Each nucleotide is mapped to a token embedding, forming an embedding matrix of size *sequence length* × *embedding dimension*. Pooling reduces this matrix to a fixed-size vector representation.

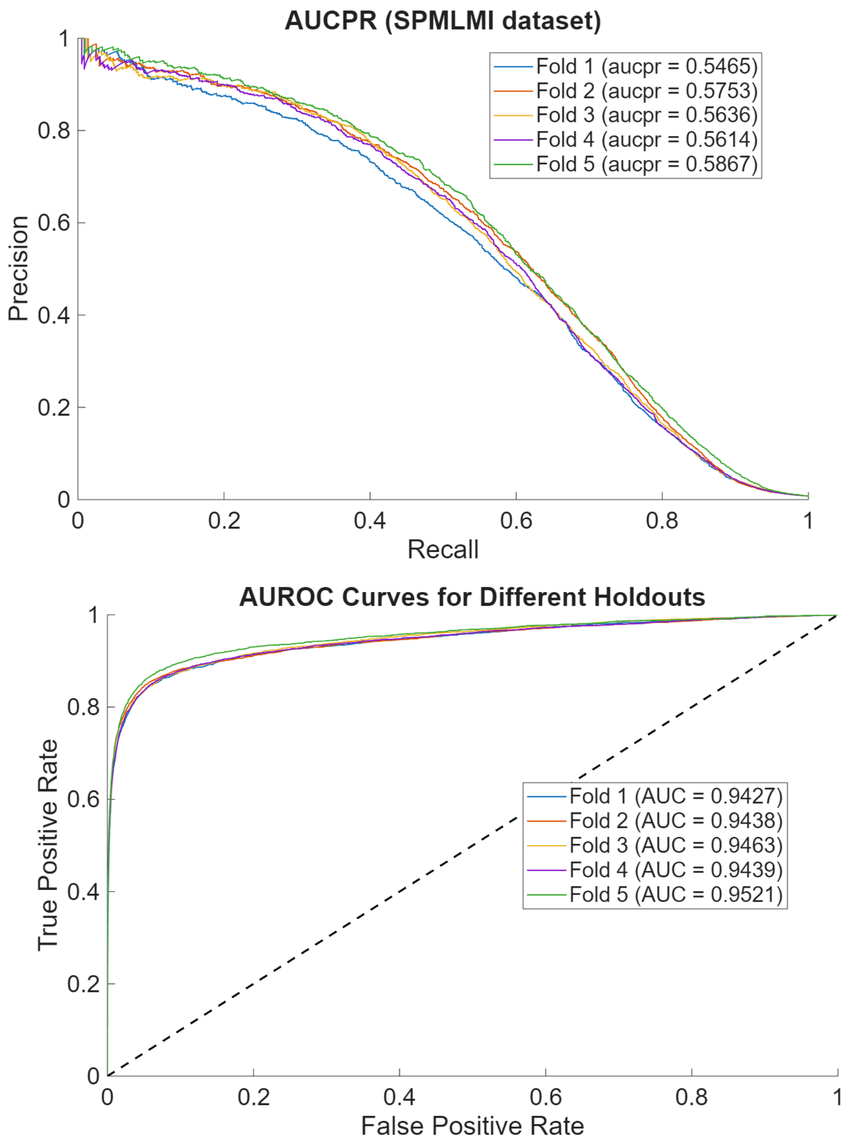


**Figure S8** AUPRC (left) and AUROC (right) results achieved by SPMLMI across the five folds on the lncRNASNP dataset.

# Tables

**Table S1.** Performance (mean $\pm$ $\sigma$) of miRInter-Trans with the three pooling strategies on the RNA-KG dataset, where $\sigma$ denotes the *sample* standard deviation across the five folds. Reported $p$-values correspond to one-sided paired $t$-tests (the best strategy - miRInter-Trans-Concat - with the remaining methods) for AUROC and AUPRC across the three interaction types. Significant $p$-values ($p < 0.05$) are in bold.

| Interaction Type | Method | AUROC (mean $\pm$ $\sigma$) | AUPRC (mean $\pm$ $\sigma$) | $p_{\text{AUROC}}$ | $p_{\text{AUPRC}}$ |
|---|---|---|---|---|---|
| miRNA–lncRNA | miRInter-Trans-Concat | **0.932 $\pm$ 0.002** | **0.334 $\pm$ 0.010** | - | - |
| | miRInter-Trans-AVG | 0.927 $\pm$ 0.001 | 0.311 $\pm$ 0.004 | **0.00094** | **0.00246** |
| | miRInter-Trans-MAX | 0.931 $\pm$ 0.001 | 0.331 $\pm$ 0.003 | 0.11384 | 0.24256 |
| | miRInter-Trans-Concat-noAUG | 0.8887 $\pm$ 0.0035 | 0.2383 $\pm$ 0.0046 | - | - |
| | miRInter-Trans-AVG-noAUG | 0.8828 $\pm$ 0.002 | 0.2212 $\pm$ 0.0052 | - | - |
| | miRInter-Trans-MAX-noAUG | 0.8867 $\pm$ 0.0034 | 0.2338 $\pm$ 0.0094 | - | - |
| miRNA-miRNA | miRInter-Trans-Concat | **0.878 $\pm$ 0.007** | **0.375 $\pm$ 0.015** | - | - |
| | miRInter-Trans-AVG | 0.870 $\pm$ 0.004 | 0.354 $\pm$ 0.012 | **0.01172** | **0.01125** |
| | miRInter-Trans-MAX | 0.871 $\pm$ 0.009 | 0.364 $\pm$ 0.022 | **0.01835** | 0.05076 |
| | miRInter-Trans-Concat-noAUG | 0.8659 $\pm$ 0.0103 | 0.3289 $\pm$ 0.0320 | - | - |
| | miRInter-Trans-AVG-noAUG | 0.8566 $\pm$ 0.01 | 0.3339 $\pm$ 0.0119 | - | - |
| | miRInter-Trans-MAX-noAUG | 0.8627 $\pm$ 0.0056 | 0.3315 $\pm$ 0.0233 | - | - |
| miRNA-snoRNA | miRInter-Trans-Concat | **0.931 $\pm$ 0.002** | **0.465 $\pm$ 0.010** | - | - |
| | miRInter-Trans-AVG | 0.926 $\pm$ 0.003 | 0.445 $\pm$ 0.008 | **0.00066** | **0.00727** |
| | miRInter-Trans-MAX | 0.927 $\pm$ 0.003 | 0.450 $\pm$ 0.004 | **0.00067** | **0.02467** |
| | miRInter-Trans-Concat-noAUG | 0.909 $\pm$ 0.0057 | 0.3744 $\pm$ 0.0282 | - | - |
| | miRInter-Trans-AVG-noAUG | 0.9004 $\pm$ 0.0018 | 0.3711 $\pm$ 0.0188 | - | - |
| | miRInter-Trans-MAX-noAUG | 0.9065 $\pm$ 0.0092 | 0.3673 $\pm$ 0.0343 | - | - |

**Table S2.** Performance (mean $\pm$ $\sigma$) of miRInter-Trans-Concat compared with IntaRNA and Random baseline for AUROC and AUPRC on the RNA-KG dataset, where $\sigma$ denotes the *sample* standard deviation across the five folds. Reported $p$-values correspond to one-sided paired $t$-tests (miRInter-Trans-Concat with the remaining methods). Significant $p$-values ($p < 0.05$) are in bold.

| Interaction Type | Method | AUROC (mean $\pm$ $\sigma$) | AUPRC (mean $\pm$ $\sigma$) | $p_{\text{AUROC}}$ | $p_{\text{AUPRC}}$ |
|---|---|---|---|---|---|
| miRNA–lncRNA | miRInter-Trans-Concat | **0.933 $\pm$ 0.002** | **0.334 $\pm$ 0.010** | – | – |
| | IntaRNA | 0.582 $\pm$ 0.005 | 0.048 $\pm$ 0.001 | **7.9E-9** | **1.9E-7** |
| | Random | 0.500 $\pm$ 0.000 | 0.047 $\pm$ 0.000 | **1.0E-10** | **2.1E-7** |
| miRNA–miRNA | miRInter-Trans-Concat | **0.878 $\pm$ 0.007** | **0.375 $\pm$ 0.015** | – | – |
| | IntaRNA | 0.622 $\pm$ 0.016 | 0.100 $\pm$ 0.013 | **4.3E-6** | **2.4E-7** |
| | Random | 0.500 $\pm$ 0.000 | 0.047 $\pm$ 0.000 | **1.4E-8** | **5.3E-7** |
| miRNA–snoRNA | miRInter-Trans-Concat | **0.931 $\pm$ 0.002** | **0.465 $\pm$ 0.010** | – | – |
| | IntaRNA | 0.656 $\pm$ 0.009 | 0.085 $\pm$ 0.004 | **3.2E-7** | **4.4E-8** |
| | Random | 0.500 $\pm$ 0.000 | 0.047 $\pm$ 0.000 | **8.1E-11** | **4.1E-8** |

**Table S3.** Performance in the *de novo* experimental setting on the RNA-KG dataset for the three pooling strategies of *miRInter-Trans*. Values are single-test results (no standard deviation). Best value per interaction and metric is in bold.

| Interaction Type | Method | AUROC | AUPRC |
|---|---|---|---|
| miRNA–lncRNA | miRInter-Trans-Concat | **0.879** | 0.278 |
| | miRInter-Trans-AVG | 0.878 | **0.282** |
| | miRInter-Trans-MAX | 0.869 | 0.251 |
| miRNA–miRNA | miRInter-Trans-Concat | 0.826 | 0.301 |
| | miRInter-Trans-AVG | **0.835** | **0.320** |
| | miRInter-Trans-MAX | 0.816 | 0.291 |
| miRNA–snoRNA | miRInter-Trans-Concat | 0.830 | 0.261 |
| | miRInter-Trans-AVG | **0.850** | **0.283** |
| | miRInter-Trans-MAX | 0.772 | 0.232 |

**Table S4.** Performance on the lncRNASNP data set with the 5-fold cross-validation and "de novo" experimental settings. In the 5-fold cross-validation setting, values are mean performance across folds ± standard deviation; the suffix noAUG refers to an experimental setting with no augmented data.

| Exp. setting | Approach | AUPRC(sd) / AUROC(sd) |
|---|---|---|
| 5-fold CV | miRInter-Trans-concat | **0.948** ± 0.001 / **0.987** ± 0.002 |
| | miRInter-Trans-AVG | 0.941 ± 0.003 / 0.985 ± 0.003 |
| | miRInter-Trans-MAX | 0.945 ± 0.003 / 0.987 ± 0.001 |
| | miRInter-Trans-concat-noAUG | 0.894 ± 0.006 / 0.976 ± 0.001 |
| | miRInter-Trans-AVG-noAUG | 0.875 ± 0.008 / 0.972 ± 0.003 |
| | miRInter-Trans-MAX-noAUG | 0.901 ± 0.006 / 0.977 ± 0.001 |
| "de novo" prediction | miRInter-Trans-concat | **0.784** / **0.885** |
| | miRInter-Trans-AVG | 0.778 / 0.875 |
| | miRInter-Trans-MAX | 0.775 / 0.879 |
| | miRInter-Trans-concat-noAUG | 0.431 / 0.764 |
| | miRInter-Trans-AVG-noAUG | 0.442 / 0.765 |
| | miRInter-Trans-MAX-noAUG | 0.438 / 0.743 |

**Table S5.** Performance (mean ± $\sigma$) of miRInter-Trans with the three pooling strategies on the lncRNASNP dataset, where $\sigma$ denotes the *sample* standard deviation across the five folds. Reported $p$-values correspond to one-sided paired $t$-tests (miRInter-Trans-Concat vs the remaining poolings). Significant $p$-values ($p < 0.05$) are in bold.

| Interaction Type | Method | AUROC (mean ± $\sigma$) | AUPRC (mean ± $\sigma$) | $p_{\text{AUROC}}$ | $p_{\text{AUPRC}}$ |
|---|---|---|---|---|---|
| miRNA–lncRNA | miRInter-Trans-Concat | **0.987 ± 0.001** | **0.948 ± 0.002** | – | – |
| | miRInter-Trans-AVG | 0.985 ± 0.001 | 0.942 ± 0.003 | **9.5E-4** | **8.04E-3** |
| | miRInter-Trans-MAX | 0.987 ± 0.001 | 0.945 ± 0.004 | 0.217 | **2.33E-2** |

**Table S6.** Performance (mean ± $\sigma$) of miRInter-Trans-Concat compared with SPMLMI and Random baseline on the lncRNASNP dataset, where $\sigma$ denotes the *sample* standard deviation across the five folds. Reported $p$-values correspond to one-sided paired $t$-tests (miRInter-Trans-Concat vs the other methods). Significant $p$-values ($p < 0.05$) are in bold.

| Interaction Type | Method | AUROC (mean ± $\sigma$) | AUPRC (mean ± $\sigma$) | $p_{\text{AUROC}}$ | $p_{\text{AUPRC}}$ |
|---|---|---|---|---|---|
| miRNA–lncRNA | miRInter-Trans-Concat | **0.987 ± 0.001** | **0.948 ± 0.002** | – | – |
| | SPMLMI | 0.946 ± 0.004 | 0.567 ± 0.015 | **7.09E-6** | **2.48E-7** |
| | Random | 0.500 ± 0.000 | 0.037 ± 0.000 | **5.03E-13** | **6.70E-12** |
